# A lncRNA identifies *Irf8* enhancer element in negative feedback control of dendritic cell differentiation

**DOI:** 10.7554/eLife.83342

**Published:** 2023-03-14

**Authors:** Huaming Xu, Zhijian Li, Chao-Chung Kuo, Katrin Götz, Thomas Look, Marcelo AS de Toledo, Kristin Seré, Ivan G Costa, Martin Zenke

**Affiliations:** 1 Department of Cell Biology, Institute of Biomedical Engineering, RWTH Aachen University Medical School Aachen Germany; 2 Helmholtz Institute for Biomedical Engineering, RWTH Aachen University Aachen Germany; 3 Institute for Computational Genomics, RWTH Aachen University Medical School Aachen Germany; 4 Department of Hematology, Oncology, Hemostaseology, and Stem Cell Transplantation, Faculty of Medicine, RWTH Aachen University Aachen Germany; Agency for Science Technology and Research Singapore; Indian Institute of Science Education and Research (IISER) India

**Keywords:** interferon regulatory factor 8, dendritic cell, enhancer, lncRNA, feedback loop, CRISPR, Mouse

## Abstract

Transcription factors play a determining role in lineage commitment and cell differentiation. Interferon regulatory factor 8 (IRF8) is a lineage determining transcription factor in hematopoiesis and master regulator of dendritic cells (DC), an important immune cell for immunity and tolerance. IRF8 is prominently upregulated in DC development by autoactivation and controls both DC differentiation and function. However, it is unclear how *Irf8* autoactivation is controlled and eventually limited. Here, we identified a novel long non-coding RNA transcribed from the +32 kb enhancer downstream of *Irf8* transcription start site and expressed specifically in mouse plasmacytoid DC (pDC), referred to as *lncIrf8*. The *lncIrf8* locus interacts with the *lrf8* promoter and shows differential epigenetic signatures in pDC versus classical DC type 1 (cDC1). Interestingly, a sequence element of the *lncIrf8* promoter, but not *lncIrf8* itself, is crucial for mouse pDC and cDC1 differentiation, and this sequence element confers feedback inhibition of *Irf8* expression. Taken together, in DC development *Irf8* autoactivation is first initiated by flanking enhancers and then second controlled by feedback inhibition through the *lncIrf8* promoter element in the +32 kb enhancer. Our work reveals a previously unrecognized negative feedback loop of *Irf8* that orchestrates its own expression and thereby controls DC differentiation.

## Introduction

Lineage-determining transcription factors (TF) are master regulators of gene programs that frequently initiate self-reinforcing loops by autoactivation. TF autoactivation is important for cells to pass restriction points during development (referred to as points of no return) and to enforce cellular identity. Molecular circuitries of autoactivation have been studied for several TF, such as GATA-binding factor 1 (GATA1), PU.1 (encoded by Spi1), CCAAT enhancer-binding protein α and ε (C/EBPα and ε; Graf and Enver, 2009; Loughran et al., 2020; Nishimura et al., 2000; Okuno et al., 2005; Theilgaard-Mönch et al., 2022). A further example is interferon regulatory factor 8 (IRF8), which shows autoactivation in cooperation with basic leucine zipper ATF-like transcription factor 3 (BATF3; Anderson et al., 2021; Grajales-Reyes et al., 2015). An important principle in nature is negative feedback control to avoid signal overshooting and toxicity. Negative feedback control applies also to lineage-determining TF; however, there is a paucity on our knowledge of the molecular mechanisms involved.

IRF8 is a hematopoietic TF positioned at the center of the regulatory gene network for dendritic cell (DC) development (Anderson et al., 2021; Belz and Nutt, 2012; Chauvistré and Seré, 2020; Kim et al., 2020; Lin et al., 2015; Nutt and Chopin, 2020; Tamura et al., 2015; Verlander et al., 2022). IRF8 is a member of the interferon regulatory factor (IRF) family of TF. Initially members of this TF family were found to mediate the induction of interferon induced genes, but are now known to serve diverse functions in regulating the immune system (Honda and Taniguchi, 2006; Tamura et al., 2008). *Irf8* knockout mice show abnormal development of classical DC type 1 (cDC1) and plasmacytoid DC (pDC) (Durai et al., 2019; Schiavoni et al., 2002; Sichien et al., 2016; Tsujimura et al., 2003). *Irf8* is prominently upregulated during DC development by autoactivation (Grajales-Reyes et al., 2015; Lin et al., 2015), yet how *Irf8* autoactivation is controlled and eventually limited, and the epigenetic mechanisms involved is largely unknown.

*Irf8* expression in hematopoietic cells is induced and maintained by enhancers located at –50 kb,+32 kb,+41 kb and +56 kb relative to *Irf8* transcription start site (TSS) (Anderson et al., 2021; Bagadia et al., 2019; Durai et al., 2019; Grajales-Reyes et al., 2015; Murakami et al., 2021; Schönheit et al., 2013). Enhancers are *cis*-regulatory sequences with multiple TF binding sites that cooperatively bind TF and thereby activate transcription, as demonstrated by many studies including our work (Davidson et al., 1986; Long et al., 2016; Wildeman et al., 1986; Zenke et al., 1986). Enhancers regulate complex gene networks and can also produce non-coding RNA, referred to as enhancer RNA (eRNA). eRNA serve as an indicator for enhancer activity and some eRNA have an activity on their own and act in cis or trans to regulate cell fate decisions (Sartorelli and Lauberth, 2020; Statello et al., 2021). Enhancer-associated long non-coding RNA (lncRNA) represent a class of lncRNA transcribed from active enhancers. Thus, eRNA and enhancer-associated lncRNA provide opportunities to detect enhancer activity and to investigate enhancer function.

DC are highly specialized immune cells that play a critical role in regulating innate and adaptive immune responses (Cabeza-Cabrerizo et al., 2021). DC develop from hematopoietic stem cells (HSC) via successive steps of lineage commitment and differentiation. More specifically, HSC develop into multipotent progenitors (MPP) that are committed to DC restricted common DC progenitors (CDP) and differentiate into classic DC (cDC) type 1 and type 2 (cDC1 and cDC2, respectively) and pDC (Anderson et al., 2021; Cabeza-Cabrerizo et al., 2021; Ginhoux et al., 2022; Nutt and Chopin, 2020; Rodrigues and Tussiwand, 2020). pDC were recently also shown to develop from lymphoid progenitors (Dress et al., 2019; Rodrigues et al., 2018; Rodrigues and Tussiwand, 2020). Differential expression of *Irf8* regulates DC and monocyte specification in a dose-dependent manner (Cytlak et al., 2020; Murakami et al., 2021). *Irf8* expression starts at the CDP stage, and is high in pDC and cDC1, which is attributed to the autoactivation of *Irf8* during DC subsets specification (Grajales-Reyes et al., 2015; Lin et al., 2015). Interestingly, IRF8 can act as a transcriptional activator or repressor in hematopoiesis by interacting with different partner TF and binding to specific DNA sequences (Tamura et al., 2015).

As an activator, IRF8 binds to its own promoter in DC differentiation, which is considered as the autoactivation capacity of *Irf8* (Grajales-Reyes et al., 2015; Lin et al., 2015). For instance, IRF8 interacts with partner TF, such as PU.1, to initiate *Irf8* autoactivation at the CDP stage (Grajales-Reyes et al., 2015). Inversely, IRF8 inhibits C/EBPα activity in neutrophil differentiation (Kurotaki et al., 2014). IRF8 also represses C/EBPβ to generate and maintain DC lineage-specific enhancer landscapes (Bornstein et al., 2014). In addition, IRF8 is important for the *Myc-Mycl* transition in DC differentiation (Anderson lll et al., 2021). IRF8 represses *Myc* expression in progenitors, while IRF8 at high levels interacts with PU.1 and drives *Mycl* expression (Anderson lll et al., 2021). All this emphasizes the central position of IRF8 in coordinating the gene network that regulates DC differentiation and function.

During DC differentiation, the *Irf8* gene locus shows high epigenetic dynamics, including histone modifications and TF binding identified by ChIP-seq (Chauvistré and Seré, 2020; Durai et al., 2019; Grajales-Reyes et al., 2015; Lin et al., 2015), chromatin accessibility measured by ATAC-seq (Kurotaki et al., 2019; Li et al., 2019), and three-dimensional chromatin structure remodeling determined by chromosome conformation capture (3 C) (Kurotaki et al., 2022; Schönheit et al., 2013). All this emphasizes the impact of epigenetic regulators on *Irf8* gene activity in DC differentiation. Notably, *Irf8* is flanked by multiple enhancers at –50 kb,+32 kb,+41 kb, and +56 kb that regulate *Irf8* expression in hematopoietic cells (Anderson et al., 2021; Murakami et al., 2021). These four enhancers were found to be driven by PU.1, BATF3, E proteins and Runt-related transcription factor (RUNX)-core binding factor beta (CBFβ) (RUNX-CBFβ), respectively (Bagadia et al., 2019; Durai et al., 2019; Grajales-Reyes et al., 2015; Murakami et al., 2021; Schönheit et al., 2013).

Chromatin conformation, particularly enhancer promoter interactions, provides a platform for TF-driven gene regulation and serves as a driving force for cell-fate determinations (Misteli and Finn, 2021; Oudelaar and Higgs, 2021; Stadhouders et al., 2019). Schönheit et al. demonstrated *Irf8* promoter interactions with its upstream enhancers by quantitative 3 C (Schönheit et al., 2013). In this study PU.1 was found to regulate chromatin remodeling between the –50 kb enhancer and the *Irf8* promoter in myeloid differentiation. In a recent study Kurotaki et al., 2022 determined the higher-order chromatin structure in DC progenitors, cDC1 and cDC2 on a genome-wide scale by Hi-C. In this study, reorganization of chromatin conformation at DC-specific gene loci was observed during cDC differentiation, and IRF8 was found to promote chromatin activation in DC progenitors leading to cDC lineage-specific gene expression. However, high resolution maps of the physical chromatin interactions of the *Irf8* promoter with upstream and downstream enhancers in the full complement of DC subsets, including pDC, are required for understanding *Irf8* regulation during DC differentiation.

Frequently, chromatin data, including ATAC-seq and/or ChIP-seq data, are used to identify regulatory elements of gene transcription. Here we embarked on a different approach and searched for lncRNA, which by themselves might have regulatory functions or are indicative of enhancer activity. We identified a novel lncRNA transcribed from the *Irf8* +32 kb enhancer, which is specifically expressed in pDC, referred to as *lncIrf8*. We found that the *lncIrf8* promoter element but not *lncIrf8* itself impacts pDC and cDC1 development. Thus, *lncIrf8* acts as an indicator for the *Irf8* +32 kb enhancer activity. Importantly, our study revealed a previously unrecognized negative feedback loop of *Irf8* in DC differentiation. *Irf8* first activates its expression by autoactivation via the +32 kb enhancer and second limits its own expression through the *lncIrf8* promoter element in the +32 kb enhancer.

## Results

### *lncIrf8* marks a pDC-specific *Irf8* enhancer element

**Figure 1.**
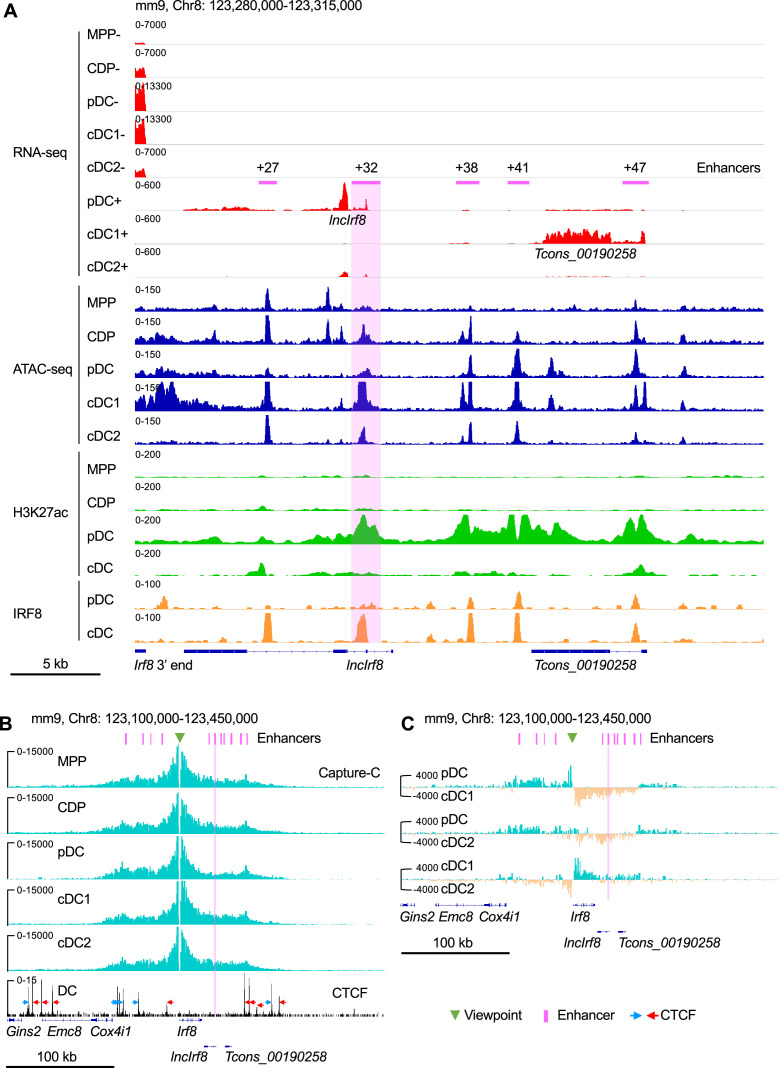
*Irf8* epigenetic signatures and promoter-enhancer interaction maps during DC differentiation. (**A**) Gene expression and epigenetic signatures of *Irf8* downstream region in MPP, CDP, pDC, all cDC, cDC1, and cDC2 are visualized by IGV browser. Gene expression was measured by RNA-seq, chromatin accessibility by ATAC-seq, H3K27ac and IRF8 binding by ChIP-seq. Positions of *Irf8* 3’ end, *Irf8* enhancers, pDC specific *lncIrf8* and cDC1 specific *Tcons_00190258* lncRNA are indicated. For RNA-seq - and +strands are shown. Scale bar: 5 kb. (**B**) Physical interactions of *Irf8* promoter with flanking sequences in MPP, CDP, pDC, cDC1, and cDC2 by nuclear-titrated (NuTi) Capture-C (turquoise), and CTCF binding by ChIP-seq in DC (Garber et al., 2012). Mean numbers of unique interactions normalized to a 300 kb region around the *Irf8* promoter viewpoint (green triangle) and scaled by a factor of 1,000,000 are shown (n=2). The orientations of CTCF binding are indicated with blue and red arrows. *Tcons_00190258* refers to the cDC1-specific lncRNA shown in (**A**). Scale bar: 100 kb. (**C**) Comparations of the chromatin interactions with *Irf8* promoter in pDC, cDC1 and cDC2. Differential tracks were created by subtraction of the mean normalized tracks of (**B**). Pairwise comparisons are shown and color coded. Turquoise and orange tracks represent specific interactions with the *Irf8* promoter in the indicated cell types. Scale bar: 100 kb. Purple bars and lines indicate the position of flanking enhancers relative to *Irf8* TSS. The purple bars from left to right represent –50 kb, –34 kb, –26 kb, –16 kb,+27 kb,+32 kb,+38 kb,+41 kb,+47 kb,+56 kb and +62 kb enhancer, respectively (panels B and C). *Irf8* +32 kb enhancer is highlighted by purple box.

*Irf8* expression in DC development is subject to complex epigenetic regulation. Here, we used an integrated approach with RNA-seq, ATAC-seq, ChIP-seq and Capture-C to track the dynamics of gene expression, histone modification and chromatin conformation in the sequel MPP, CDP, pDC, cDC1, and cDC2 (Figure 1, Figure 1—figure supplements 1 and 2).

**Figure 1—figure supplement 1.**
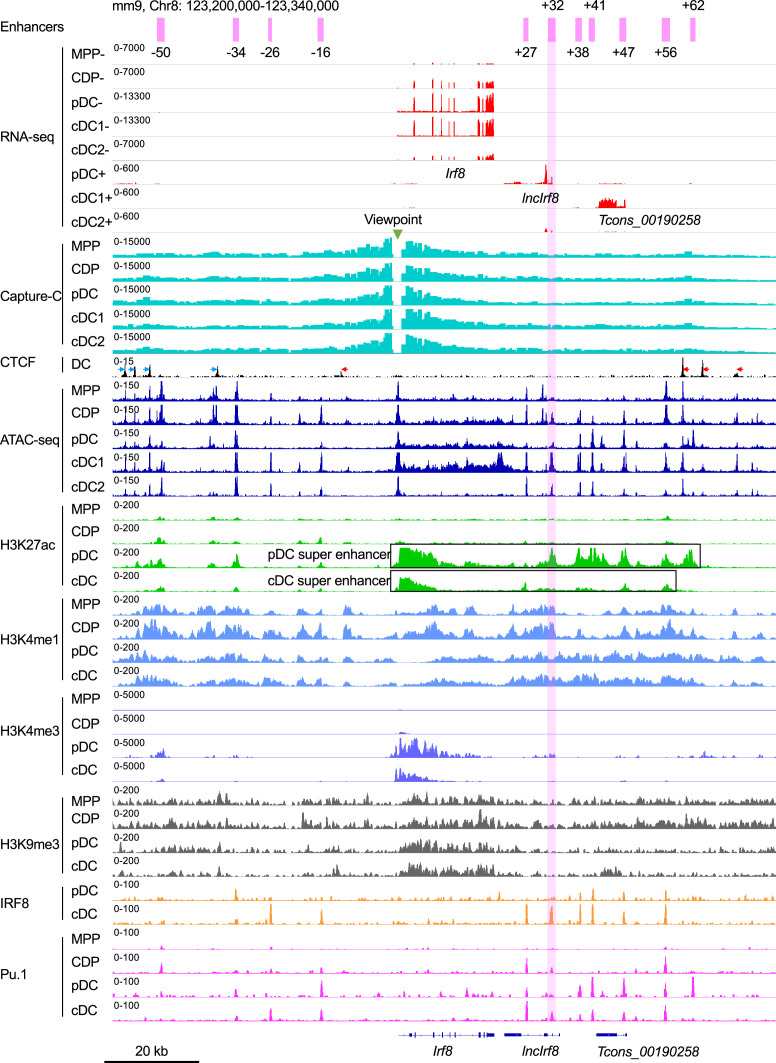
Epigenetic signatures of *Irf8* locus during DC differentiation. Gene expression and epigenetic signatures of *Irf8* gene and its flanking regions in MPP, CDP, pDC, all cDC, cDC1, and cDC2 are visualized by IGV browser. Gene expression by RNA-seq, chromatin interactions with *Irf8* promoter by Capture-C, chromatin accessibility by ATAC-seq, histone modification (H3K27ac, H3K4me3, H3K4me1, H3K9me3) and TF binding (CTCF, IRF8 and PU.1) by ChIP-seq are as in Figure 1A and B. Positions of upstream and downstream *Irf8* enhancers are in purple, Capture-C viewpoint (green triangle), *Irf8* gene, pDC-specific *lncIrf8* and cDC1 specific *Tcons_00190258* lncRNA are indicated. The orientation of CTCF binding is indicated with blue and red arrows. pDC and cDC super enhancers (Grajales-Reyes et al., 2015) are indicated with black boxes. For RNA-seq – and +strands are shown. Scale bar: 20 kb.

**Figure 1—figure supplement 2.**
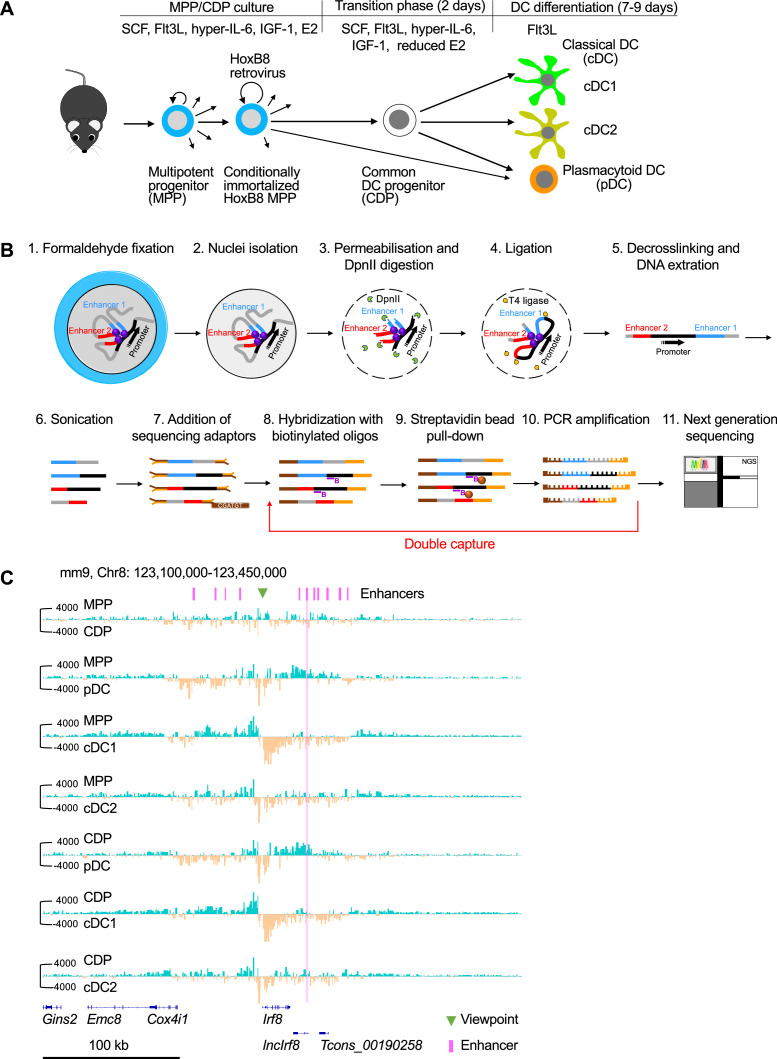
In vitro DC differentiation of HoxB8 MPP and nuclear-titrated (NuTi) Capture-C. (**A**) HoxB8 MPP were obtained by infection of bone marrow MPP with HoxB8 retrovirus and cultured in growth medium with SCF, Flt3L, hyper-IL-6, IGF-1, and β-estradiol (E2) (MPP/CDP culture). DC differentiation is induced in growth medium plus high concentration of Flt3L and reduced E2 for 2 days (transition phase). Then growth factors and E2 are removed and cells are further differentiated into DC with high concentration of Flt3L only (DC differentiation). (**B**) For nuclear-titrated (NuTi) Capture-C FACS sorted cells were fixed with formaldehyde and nuclei were isolated (step 1 and 2). Nuclei were permeabilized with SDS and digested with DpnII (step 3). DNA fragments in nuclei were re-ligated by T4 ligase (step 4) and DNA was extracted to obtain 3C libraries (step 5). The 3C libraries were subjected to sonication (~200 bp fragments), sequencing adaptors were added and DNA was then hybridized with 70 bp biotinylated oligos of the viewpoint, followed by streptavidin bead pull-down and PCR amplification (steps 6–10). Steps 8–10 were repeated to increase capture efficiency. Finally, the captured DNA was subjected to next-generation sequencing (step 11). This scheme was adapted from Figure 1A of Downes et al., 2022. (**C**) Comparison of the chromatin interactions with the *Irf8* promoter by Capture-C (viewpoint, green triangle) between MPP, CDP, pDC, cDC1, and cDC2. Positions of *Irf8* enhancers are indicated (purple). Pairwise comparisons are shown and color coded (turquoise vs orange) as in Figure 1C. Differential tracks were created by subtraction of the mean normalized tracks of Figure 1B. Scale bar: 100 kb.

**Figure 1—figure supplement 3.**
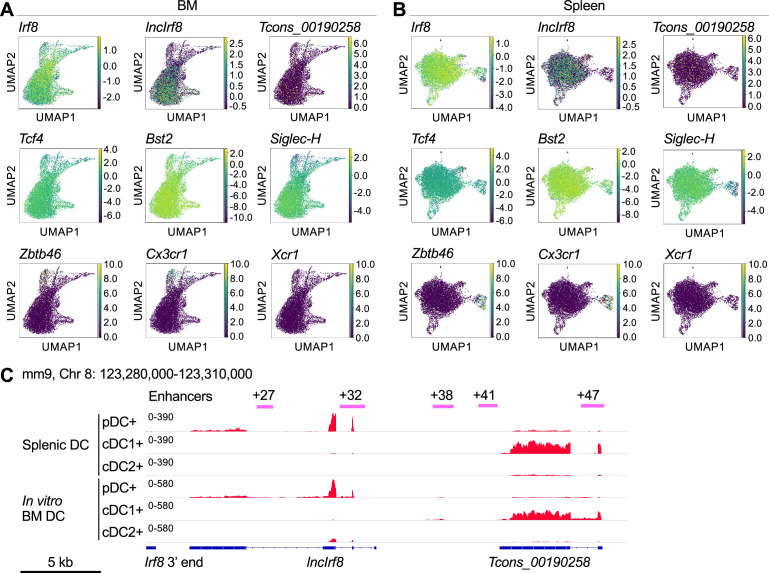
pDC specific *lncIrf8* and cDC1 specific *Tcons_00190258* lncRNA expression in ex-vivo DC. (**A and B**) UMAPs show the expression of *lncIrf8* and *Tcons_00190258* lncRNA and the indicated marker genes in BM and splenic pDC (Lin^-^ CD11c^+^ Bst2^+^ Siglec-H^+^) from scRNA-seq data (GSE114313) (Rodrigues et al., 2018). pDC marker genes, *Tcf4*, *Bst2* and *Siglec-H*; cDC marker genes, *Zbtb46*, *Cx3cr1* and *Xcr1*. (**C**) Gene expression of *lncIrf8* and *Tcons_00190258* lncRNA in splenic DC subsets in vivo and in DC subsets generated in in vitro BM Flt3L culture. Bulk RNA-seq data of splenic pDC (CD11c^int^ CD317^hi^ CD11b^-^), cDC1 (CD11c^hi^ CD317^low^ CD8^+^ CD11b^-^), and cDC2 (CD11c^hi^ CD317^low^ CD8^-^ CD11b^-^) were obtained from GSE188992 (Pang et al., 2022). The bulk RNA-seq data of DC from Flt3L culture are from this study. Gene expression is visualized by IGV browser. Positions of *Irf8* 3’ end, *Irf8* enhancers, pDC-specific *lncIrf8* and cDC1 specific *Tcons_00190258* lncRNA are indicated. Scale bar: 5 kb.

We performed de novo transcript assembly of the RNA-seq data and detected two previously unknown transcripts without coding potential downstream of *Irf8*: a pDC specific lncRNA (*Tcons_00190250*) in the following referred to as *lncIrf8* and a cDC1 specific lncRNA (*Tcons_00190258*; Figure 1A and Figure 1—figure supplement 1). *lncIrf8* and *Tcons_00190258* show the same expression pattern in pDC and cDC1, respectively, in BM and spleen (Figure 1—figure supplement 3), as revealed by reanalyzing scRNA-seq and bulk RNA-seq data (Pang et al., 2022; Rodrigues et al., 2018). *lncIrf8* is transcribed within an enhancer region located 32 kb downstream of the *Irf8* TSS labeled by H3K27ac and H3K4me1 and occupied by DC differentiation-associated TF, such as IRF8 and PU.1 (Figure 1A and Figure 1—figure supplement 1). This region is largely devoid of H3K9me3, a chromatin modification frequently associated with heterochromatin, indicating an open chromatin configureuration in DC (Figure 1—figure supplement 1). In addition, sequences of this region have been implicated in DC development and referred to as +32 kb enhancer (Durai et al., 2019). Thus, we proceeded to study *lncIrf8* in detail.

**Figure 2.**
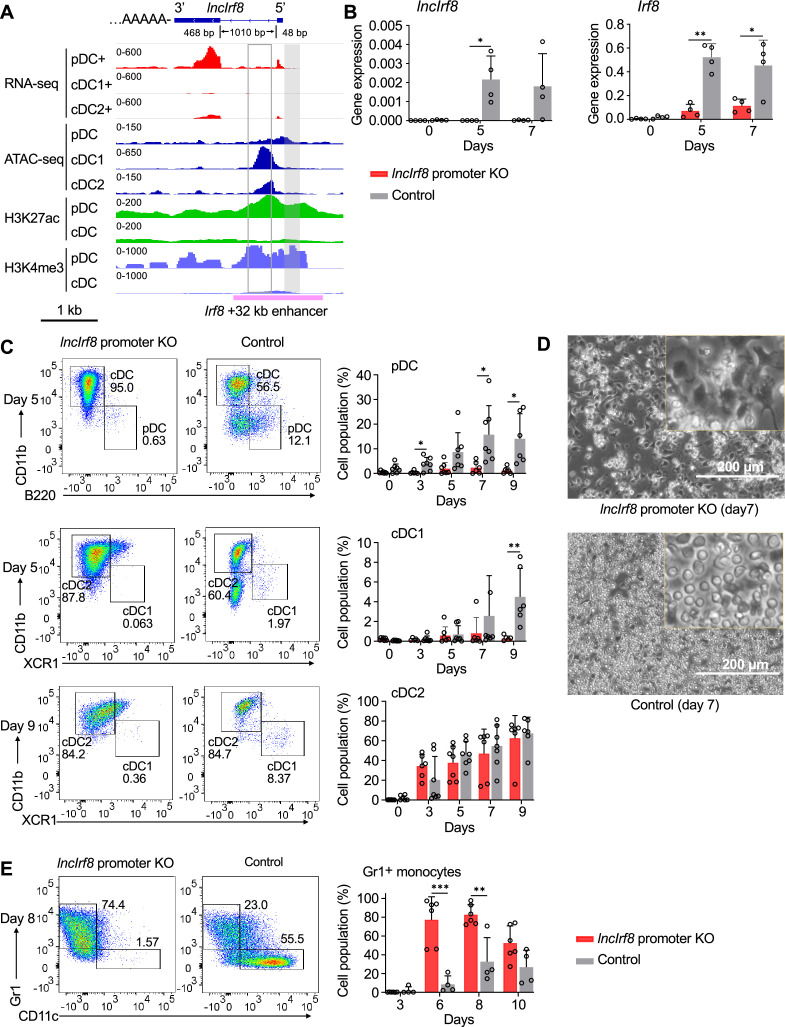
*IncIrf8* promoter KO compromises pDC and cDC1 development in vitro. (**A**) Genomic anatomy of *lncIrf8* locus determined by 3’ and 5’ RACE PCR. Blue box, exon 2 and 3 (48 bp and 468 bp, respectively). The 1010 bp intron and polyA tail are shown. Data of RNA-seq, ATAC-seq, ChIP-seq of H3K27ac (enhancer mark) and H3K4me3 (active promoter mark, near TSS) are visualized by IGV browser for the indicated cell populations (pDC, all cDC, cDC1 and cDC2). Grey box, *lncIrf8* promoter KO region; open box, cDC1 specific +32 kb enhancer by Durai et al., 2019. *Irf8* +32 kb enhancer based on the H3K27ac enhancer mark is indicated with a purple line. Scale bar: 1 kb. (**B**) Gene expression of *lncIrf8* and *Irf8* in *lncIrf8* promoter KO and control at day 0, 5, and 7 of Flt3L directed DC differentiation. Gene expression was determined by RT-qPCR and normalized to *GAPDH*. n=4. (**C**) Representative flow cytometry analysis of Flt3L directed DC differentiation of *lncIrf8* promoter KO HoxB8 MPP and control (Lutz et al., 2022; Xu et al., 2022). pDC, all cDC, cDC1, and cDC2 were gated as in Figure 2—figure supplement 1E and are shown. Bar diagrams depict quantification of pDC, cDC1 and cDC2 normalized to living single cells on DC differentiation day 0, 3, 5, 7, and 9. n=6–7. (**D**) Representative phase-contrast microscopy images of *lncIrf8* promoter KO HoxB8 MPP and control on day 7 of Flt3L directed DC differentiation. Scale bar: 200 μm. (**E**) Representative flow cytometry analysis of spontaneous DC differentiation of *lncIrf8* promoter KO HoxB8 MPP and control with growth factors but without E2 (Lutz et al., 2022; Xu et al., 2022) at day 8. Gr1^+^ monocytes and CD11c^+^ DC are shown. Quantification of Gr1^+^ monocytes of living single cells on day 3, 6, 8, and 10 of spontaneous DC differentiation. n=6, *lncIrf8* promoter KO; n=4, control. Empty gRNA vector or non-targeting gRNA vector HoxB8 MPP were used as controls. Data represent mean ± SD of at least three independent experiments with different HoxB8 MPP clones of *lncIrf8* promoter KO and control without deletion. *p<0.05, **p<0.01, ***p<0.001, multiple t-tests. Data that have no difference (p>0.05) are not labeled.

ATAC-seq analysis revealed further details of the *lncIrf8* region in CDP, pDC, cDC1 and cDC2 (Figure 1A, Figure 2A and Figure 1—figure supplement 1). In cDC1 the prominent ATAC-seq and IRF8 peaks mark the cDC1 specific +32 kb enhancer (Durai et al., 2019). In pDC the ATAC-seq peak is smaller and shifted further towards downstream but aligns well with the valley in the prominent H3K27ac peak. This ATAC-seq peak marks the *lncIrf8* promoter and aligns with p300 (Durai et al., 2019) and H3K4me3 (Figure 2A and Figure 1—figure supplement 1). All this indicates that this chromatin region is open and transcriptionally active in pDC, enabling *lncIrf8* transcription.

**Figure 2—figure supplement 1.**
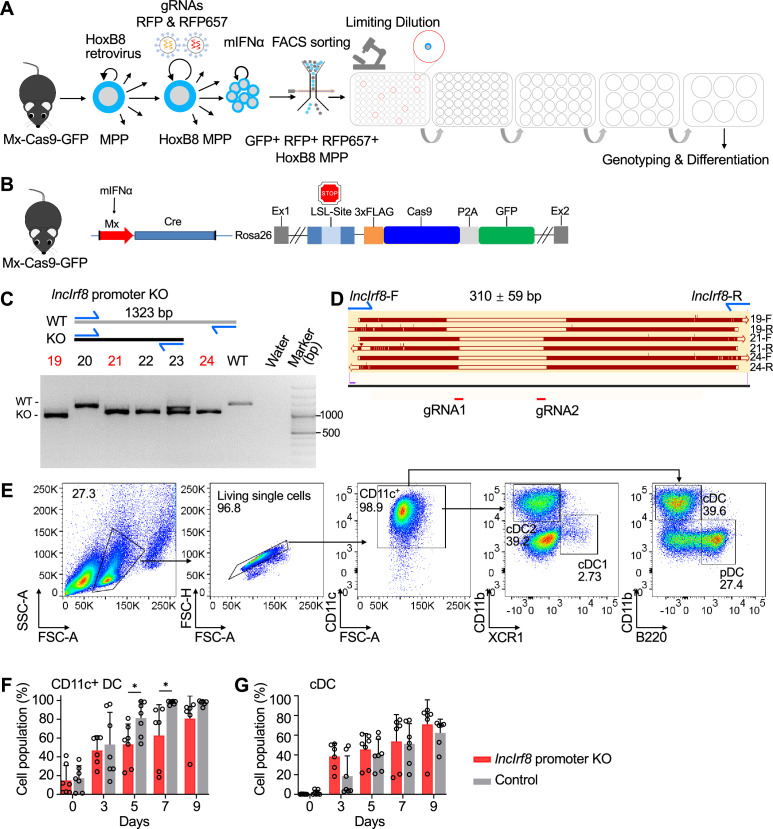
Generating *lncIrf8* promoter KO HoxB8 MPP and its impact on DC differentiation. (**A**) Mx-Cas9-GFP HoxB8 MPP were infected with 2 gRNA in RFP and RFP657 lentiviral vectors. Mouse interferon α (mIFNα) was applied for 4 hours to induce Cas9 and GFP expression. Cells were cultured for another 2 days and FACS sorted for GFP^+^ RFP^+^ RFP657^+^ cells and 3000 FACS sorted HoxB8 MPP were subjected to limiting dilution to obtain single cell colonies. Single cell HoxB8 MPP were genotyped by genomic PCR and clones with homozygous deletions in the *lncIrf8* promoter were used for further studies. (**B**) Mx-Cas9-GFP mice contain a mIFNα inducible Cre recombinase that removes the stop cassette and allows Cas9 and GFP expression. Cas9 and GFP protein are separated by the 2 A self-cleaving peptide (P2A) and thus all GFP^+^ cells express Cas9. (**C and D**) Representative genotyping of *lncIrf8* promoter KO in single-cell Mx-Cas9-GFP HoxB8 MPP. Homozygous deletions in *lncIrf8* promoter (red) were detected by PCR and agarose gel electrophoresis (**C**). *lncIrf8* promoter deletions determined by Sanger sequencing in three representative KO clones of (**C**) are depicted in (**D**). *lncIrf8*-F and *lncIrf8*-R refers to primers for genotyping and Sanger sequencing, and sequences for both directions, referred to F and R, of clones 19, 21, and 24 are shown. Positions of gRNA1 and gRNA2, which were used for generating the deletion in the *lncIrf8* promoter, are indicated. (**E**) Gating strategy for Flt3L directed DC differentiation. CD11c^+^ DC were gated on CD11b^-^ B220^+^ pDC, CD11b^+^ B220^-^ cDC, CD11b^low/-^ XCR1^+^ cDC1, and CD11b^+^ XCR1^-^ cDC2. (**F and G**) Quantification of CD11c^+^ DC and cDC of panel (**E**) of living single cells in *lncIrf8* promoter KO HoxB8 MPP and control on Flt3L directed DC differentiation at day 0, 3, 5, 7, and 9. n=6–7. Data represent mean ± SD of at least three independent experiments with different HoxB8 MPP clones of *lncIrf8* promoter KO and control. *p<0.05, multiple t-tests. Data that have no difference (p>0.05) are not labeled. Figure 2—figure supplement 1—source data 1.Full raw unedited gel of genotyping of *lncIrf8* promoter KO HoxB8 MPP clones in Figure 2—figure supplement 1C. Figure 2—figure supplement 1—source data 2.Labeled uncropped gel of genotyping of *lncIrf8* promoter KO HoxB8 MPP clones in Figure 2—figure supplement 1C.

**Figure 2—figure supplement 2.**
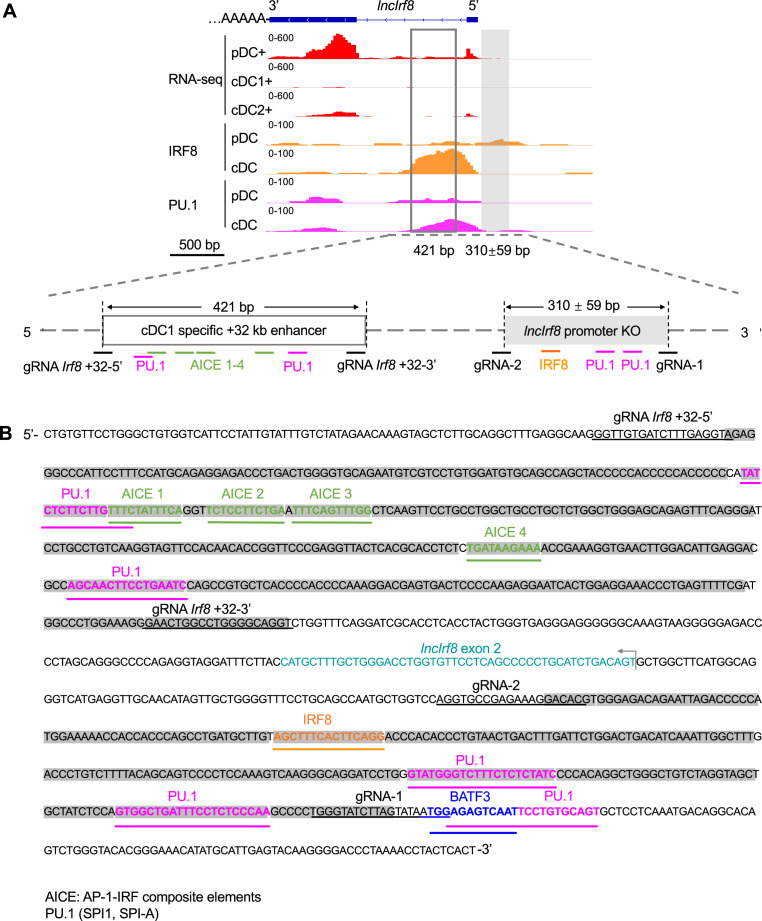
Information of *lncIrf8* promoter KO. (**A**) Schematic representation of *lncIrf8* locus. The sequences deleted in the *lncIrf8* promoter KO are indicated by a grey filled box and multiple potential IRF8 and PU.1 binding sites are depicted. The cDC1 specific +32 kb enhancer by Durai et al., 2019 is shown by an open box. Gene expression was by RNA-seq and IRF8 and PU.1 TF binding was by ChIP-seq as in Figure 1A, Figure 2A and Figure 1—figure supplement 1. Scale bar: 500 bp. (**B**) Sequence of *lncIrf8* promoter and cDC1 specific +32 kb enhancer. Sequences underlined in black indicate gRNAs used for generating the deletions. Sequence motives for AP1-IRF composite elements (AICE), IRF8, PU.1, and BATF3 were indicated with green, orange, purple, and blue lines, respectively. *lncIrf8* exon 2 is in turquoise; arrow indicates *lncIrf8* transcriptional start site and direction. The shaded sequences represent the KO region of *lncIrf8* promoter and the cDC1 specific +32 kb enhancer by Durai et al., 2019.

**Figure 2—figure supplement 3.**
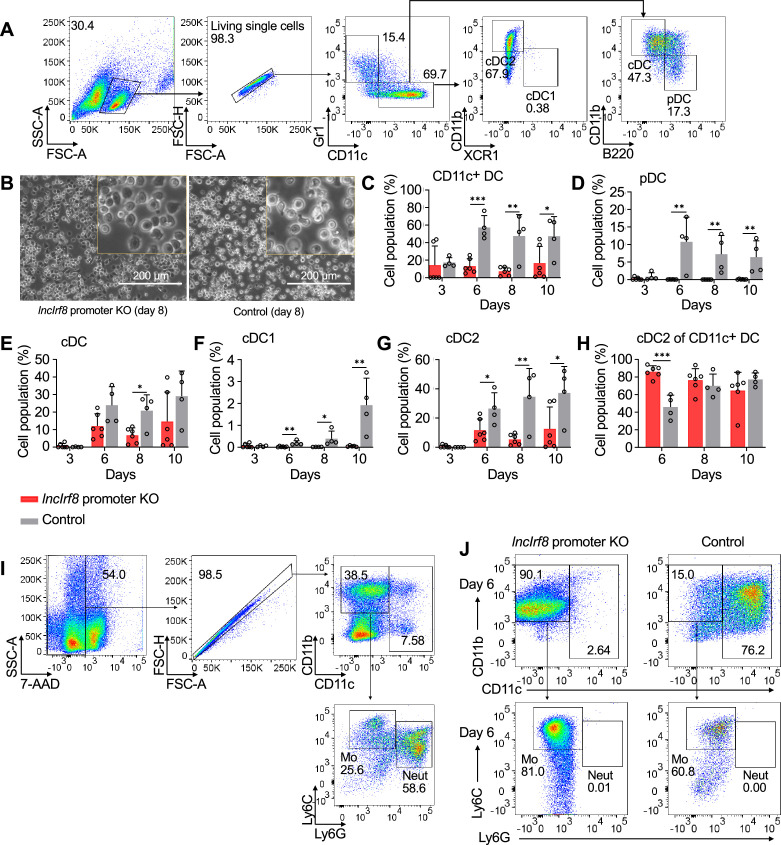
Impact of *lncIrf8* promoter KO on spontaneous DC differentiation. (**A**) Gating strategy for spontaneous DC differentiation. Gr1^+^ monocytes, and the pDC, all cDC, cDC1 and cDC2 subsets from Gr1^-^ CD11c^+^ cells were gated as in Figure 2—figure supplement 1E. (**B**) Representative phase-contrast microscopy images of *lncIrf8* promoter KO HoxB8 MPP and control on spontaneous DC differentiation (-E2) day 8. Scale bar: 200 μm. (**C–G**) Quantification of CD11c^+^ DC, pDC, all cDC, cDC1 and cDC2 of panel (**A**) of living single cells in *lncIrf8* promoter KO HoxB8 MPP and control on spontaneous DC differentiation (-E2) day 3, 6, 8, and 10. n=6 for *lncIrf8* promoter KO; n=4 for KO control. Data represent mean ± SD of at least three independent experiments with different HoxB8 MPP clones of *lncIrf8* promoter KO and control without deletion. *p<0.05, **p<0.01, ***p<0.001, multiple t-tests. Data that have no difference (p>0.05) are not labeled. (**H**) Quantification of cDC2 normalized to CD11c^+^ cells of (**G**) at days 6, 8, and 10. Spontaneous DC differentiation of control cells progressed slower than differentiation of *lncIrf8* promoter KO cells and therefore a lower frequency of cDC2 was observed at day 6. At later time points *lncIrf8* promoter KO left cDC2 differentiation unaffected. (**I**) Gating strategy for identification of Gr1^+^ monocytes upon spontaneous DC differentiation in panel (**A**) and Figure 2E. Mouse BM cells were used and Gr1^+^ monocytes (Mo) were gated as 7-AAD^-^ CD11b^+^ CD11c^-^ Ly6C^+^ Ly6G^-^. Neutrophils (Neut) were gated as 7-AAD^-^ CD11b^+^ CD11c^-^ Ly6C^low/-^ Ly6G^+^ . (**J**) Representative flow cytometry analysis of Gr1^+^ monocytes at day 6 of spontaneous DC differentiation of *lncIrf8* promoter KO and control. Cells were gated as in panel (**I**).

**Figure 2—figure supplement 4.**
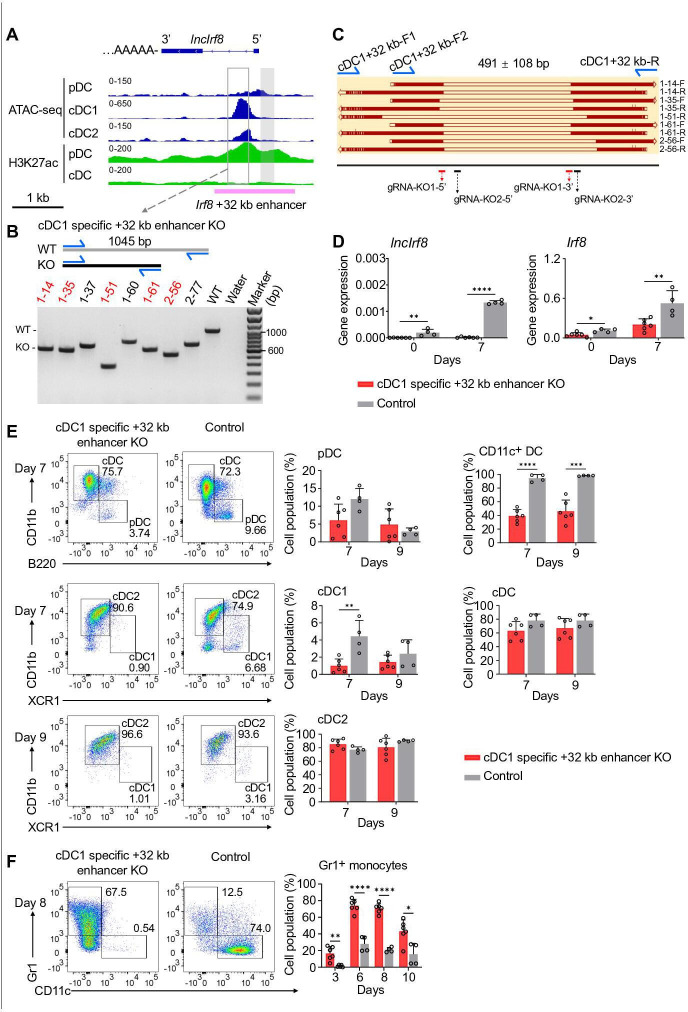
cDC1 specific +32 kb enhancer KO compromises cDC1 development in vitro. (**A**) Position of cDC1 specific +32 kb enhancer. Data of ATAC-seq, ChIP-seq of H3K27ac (enhancer mark) are visualized by IGV browser for the indicated cell populations (pDC, all cDC, cDC1, and cDC2). Open box, cDC1 specific +32 kb enhancer by Durai et al., 2019; grey box, *lncIrf8* promoter KO region; *Irf8* +32 kb enhancer based on the H3K27ac enhancer mark is indicated with a purple line. Scale bar: 1 kb. (**B and C**) Representative genotyping of cDC1 specific +32 kb enhancer KO in single-cell Mx-Cas9-GFP HoxB8 MPP. Homozygous deletions in cDC1 specific +32 kb enhancer KO (red) were detected by PCR and agarose gel electrophoresis (**B**). cDC1 specific +32 kb enhancer KO deletions determined by Sanger sequencing in five representative KO clones of (**B**) are depicted in (**C**). cDC1 +32 kb-F1 and cDC1 +32 kb-R refers to primers for genotyping. Sanger sequencing was performed from on both DNA strands: cDC1 +32 kb-F2 and cDC1 +32 kb-R was used for forward and reverse sequencing, respectively. Clones 1–14, 1–35, 1–51, 1–61, and 2–56 are shown. Positions of gRNAs used for generating the deletion of cDC1 +32 kb enhancer KO are indicated. gRNA-KO1-5' and gRNA-KO1-3' are from Durai et al., 2019; gRNA-KO2-5' and gRNA-KO2-3' were designed in the present study. (**D**) Gene expression of *lncIrf8* and *Irf8* in cDC1 specific +32 kb enhancer KO and control at day 0 and 7 of Flt3L directed DC differentiation. n=6, cDC1 specific +32 kb enhancer KO; n=4, control. (**E**) Representative flow cytometry analysis of Flt3L directed DC differentiation of cDC1 specific +32 kb enhancer KO HoxB8 MPP and control. pDC, all cDC, cDC1, and cDC2 were gated as in Figure 2—figure supplement 1E and are shown. Bar diagrams on the left depict quantification of pDC, cDC1 and cDC2 normalized to CD11c^+^ cells on DC differentiation day 7 and 9. Bar diagrams on the right depict CD11c^+^ DC and all cDC. CD11c^+^ DC were gated on living single cells. Frequencies of pDC were compromised in +32 kb enhancer KO at day 7, however this was not statistically significant. n=6, cDC1 specific +32 kb enhancer KO; n=4, control. (**F**) Representative flow cytometry analysis of spontaneous DC differentiation of cDC1 specific +32 kb enhancer KO and control with growth factors but without E2 at day 8. Gr1^+^ monocytes and CD11c^+^ DC are shown. Quantification of Gr1^+^ monocytes of living single cells on days 3, 6, 8, and 10 of spontaneous DC differentiation. n=6, cDC1 specific +32 kb enhancer KO; n=4, control. Empty gRNA vector or non-targeting gRNA vector HoxB8 MPP were used as controls. Data represent mean ± SD of three independent experiments with different HoxB8 MPP clones of cDC1 specific +32 kb enhancer KO and control without deletion. Differentiation kinetics of clones were normatized to control without deletion if required. *p<0.05, **p<0.01, ****p<0.0001, multiple t-tests. Data that have no difference (p>0.05) are not labeled. Figure 2—figure supplement 4—source data 1.Full raw unedited gel of genotyping of cDC1 specific +32 kb enhancer KO clones in Figure 2—figure supplement 4B. Figure 2—figure supplement 4—source data 2.Labeled uncropped gel of genotyping of cDC1 specific +32 kb enhancer KO clones in Figure 2—figure supplement 4B.

Next, we determined the chromatin conformation of the *Irf8* locus and the *lncIrf8* region. We generated interaction profiles by nuclear-titrated (NuTi) Capture-C in MPP, CDP, pDC, cDC1, and cDC2 (Figure 1—figure supplement 2A and B) using *Irf8* promoter as viewpoint. The *Irf8* promoter shows multiple interactions with regions spanning up to ~100 kb upstream and downstream of *Irf8* (Figure 1B and Figure 1—figure supplement 1). In pDC, the *Irf8* promoter interactions are stronger with the upstream sequences than with downstream sequences (Figure 1C and Figure 1—figure supplement 2C). In cDC1 *Irf8* promoter interactions are more confined to the regions downstream of *Irf8* compared to MPP, CDP and pDC (Figure 1C and Figure 1—figure supplement 2C). This suggests that upstream and downstream sequences of *Irf8* gene are involved in differentially regulating *Irf8* expression and controlling the development of pDC and cDC1, respectively.

The CCCTC-binding factor (CTCF) is important for regulation of chromatin conformation through loop extrusion (Sanborn et al., 2015) and we therefore visualized CTCF binding sites in the *Irf8* locus in DC (Garber et al., 2012). Interestingly, most of the *Irf8* flanking enhancers (Durai et al., 2019; Grajales-Reyes et al., 2015; Murakami et al., 2021; Schönheit et al., 2013) are located within convergent CTCF binding sites upstream and downstream of the *Irf8* gene (Figure 1B and Figure 1—figure supplement 1). There are also multiple interactions within this region without convergent CTCF binding sites, suggesting interactions with *Irf8* promoter in a CTCF independent manner, such as by TF binding, active histone modifications and gene transcription (Figure 1B and Figure 1—figure supplement 1; Owens et al., 2022).

Surprisingly, in pDC H3K27ac at the *lncIrf8* promoter is high, but this locus shows less interactions with the *Irf8* promoter in pDC compared to CDP, cDC1 and cDC2 (Figure 1C and Figure 1—figure supplement 2C). In addition, in pDC the IRF8 protein occupancy at the *lncIrf8* promoter is low and much higher in cDC (Figure 1A and Figure 1—figure supplement 1; Durai et al., 2019; Grajales-Reyes et al., 2015).

These observations warrant further studies and we thus proceeded to investigate the *lncIrf8* locus in detail.

### *lncIrf8* promoter KO compromises pDC and cDC1 development

First, we annotated *lncIrf8*. Our de-novo transcript assembly of RNA-seq data revealed different isoforms of *lncIrf8*, with the most prominent isoform comprising exon 2 and 3 (Figure 1A, Figure 2A and Figure 1—figure supplement 1). Additionally, 3' end and 5' end RACE PCR confirmed the anatomy of this *lncIrf8* isoform: two exons, one intron, and a polyA tail (Figure 2A). As expected *lncIrf8* is not conserved across species (data not shown), which is in line with the general characteristics of lncRNA. Then second, we deleted 300 bp in the *lncIrf8* promoter by CRISPR/Cas9 editing in conditionally immortalized HoxB8 MPP (Figure 2A and Figure 2—figure supplement 1A–D). The *lncIrf8* promoter is located in the *Irf8* +32 kb enhancer region and is in close proximity to the cDC1 specific +32 kb enhancer (Durai et al., 2019; Figure 2A and Figure 2—figure supplement 2). The 300 bp deletion comprises the H3K4me3 promoter mark and is confined to open chromatin identified by ATAC-seq and positioned in the valley of the H3K27ac mark (Figure 2A). Additionally, it contains binding sites for IRF8, PU.1, and BATF3 TF, which are important for DC development (Figure 2A and Figure 2—figure supplement 2B).

Generation of a precise deletion requires clonal cell populations, which is hardly achieved in somatic cells due to their limited lifespan. Therefore, we developed a Mx-Cas9-GFP system of conditionally immortalized HoxB8 MPP, which upon differentiation faithfully recapitulate DC development (Figure 2—figure supplement 1A, B; Xu et al., 2022). HoxB8 MPP were obtained from bone marrow of Mx-Cas9-GFP mice by infection with the estrogen (E2) inducible HoxB8-ER. These HoxB9 MPP exhibited an extended lifespan and robust clonogenic potential and differentiated into all DC subsets in vitro and in vivo (Xu et al., 2022). Infection of gRNA targeting *lncIrf8* promoter in Mx-Cas9-GFP HoxB8 MPP and induction of Cas9 by interferon generated single-cell *lncIrf8* promoter KO clones. Five out of 71 single-cell colonies with homozygous deletions were further studied and subjected to DC differentiation (Figure 2C–E, Figure 2—figure supplement 1C–G and Figure 2—figure supplement 3).

*lncIrf8* promoter KO abolished *lncIrf8* expression during DC differentiation compared to control without deletion (Figure 2B). Surprisingly, *Irf8* expression was also severely compromised, which points to a cross-talk of the *lncIrf8* promoter element with the *Irf8* promoter. To determine whether *lncIrf8* promoter KO also impacts DC subsets, CD11c^+^ DC, pDC and cDC subsets cDC1 and cDC2 were analyzed (Figure 2C and Figure 2—figure supplement 1E–G). Frequencies of pDC and cDC1 were severely reduced, while cDC2 were unaffected (Figure 2C). Accordingly, *lncIrf8* promoter KO cultures contained mainly cDC2 and some undifferentiated cells and were more homogenous compared to control without deletion, which contain multiple DC subsets (Figure 2C and D, Figure 2—figure supplement 1F, G).

*lncIrf8* promoter KO affected also the differentiation propensity of progenitors upon E2 withdrawal from MPP/CDP culture (Figure 1—figure supplement 2A and Figure 2—figure supplement 3). *lncIrf8* promoter KO showed a marked increase in strongly adhesive cells compared to control (Figure 2—figure supplement 3B). *lncIrf8* promoter KO cultures had higher frequencies of Gr1^+^ monocytes (Figure 2E, Figure 2—figure supplement 3I, J) and lower frequencies of all DC subsets CD11c^+^ DC, pDC, cDC1, and cDC2 (Figure 2—figure supplement 3C–G) compared to control without deletion.

The *lncIrf8* promoter element is in close proximity to the cDC1 specific +32 kb enhancer previously described in mice by Durai et al., 2019 and thus we generated the cDC1 specific +32 kb enhancer KO following the same procedure as for the *lncIrf8* promoter KO. Five out of 165 single-cell clones with homozygous deletions of cDC1 +32 kb enhancer were subjected to DC differentiation and further analyzed (Figure 2—figure supplement 4). Similar to *lncIrf8* promoter KO, cDC1 specific +32 kb enhancer KO abolished *lncIrf8* expression and also decreased *Irf8* expression during DC differentiation (Figure 2—figure supplement 4D). The cDC1 +32 kb enhancer KO also compromised the frequency of cDC1 in Flt3L directed DC differentiation, while cDC2 were unaffected (Figure 2—figure supplement 4E). Frequencies of pDC were also compromised at day 7; however, this was not statistically significant. These observations are in line with previous studies in mice that cDC1 +32 kb enhancer KO compromised cDC1 differentiation and left pDC and cDC2 largely unaffected (Durai et al., 2019; Murakami et al., 2021). In addition, cDC1 +32 kb enhancer KO cultures had higher frequencies of Gr1^+^ monocytes upon spontaneous DC differentiation by withdrawal of E2 (Figure 2—figure supplement 4F) and thus showed a similar phenotype as the *lncIrf8* promoter KO upon spontaneous DC differentiation (Figure 2E).

**Figure 3.**
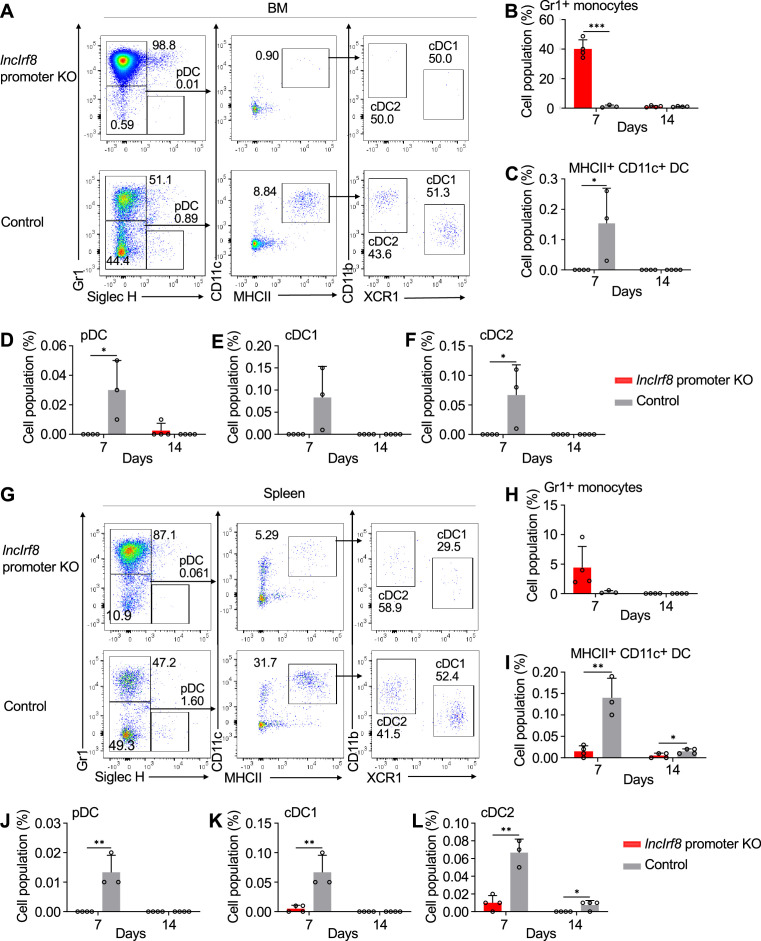
*lncIrf8* promoter KO comprises pDC and cDC1 development in vivo upon cell transplantation. (**A**) Representative flow cytometry analysis of CD45.2 *lncIrf8* promoter KO and control HoxB8 MPP in BM at day 7 after cell transplantation (for details see Figure 3—figure supplement 1A, B). Donor cell populations were gated from 7-AAD^-^ CD45.2^+^ Lin^-^ cells and Gr1^+^ monocytes, pDC, cDC1 and cDC2 are shown. (**B–F**) Quantification of Gr1^+^ monocytes, MHCII^+^ CD11c^+^ DC, pDC, cDC1, and cDC2 of living single cells in BM on day 7 and 14 after cell transplantation (n=3–4). (**G**) Representative flow cytometry analysis of *lncIrf8* promoter KO and control HoxB8 MPP in spleen at day 7 after cell transplantation. Gating was as in panel (**A**). (**H–L**) Quantification of Gr1^+^ monocytes, MHCII^+^ CD11c^+^ DC, pDC, cDC1 and cDC2 on day 7 and 14 after cell transplantation (n=3–4). Data represent mean ± SD from 3 to 4 mice. *p<0.05, **p<0.01, ***p<0.001, multiple t-tests. Data that have no difference (p>0.05) are not labeled.

Given the novel phenotype of the *lncIrf8* promoter KO, we proceeded to investigate the impact of the *lncIrf8* promoter KO on DC differentiation in vivo in mice. We transplanted CD45.2 *lncIrf8* promoter KO and CD45.2 control HoxB8 MPP into irradiated CD45.1 recipient mice (Figure 3—figure supplement 1A). DC in bone marrow and spleen were analyzed by flow cytometry on day 7 and 14 after cell transplantation (Figure 3, Figure 3—figure supplement 1A, B). In bone marrow, *lncIrf8* promoter KO cells mostly differentiated into Gr1^+^ monocytes, and lower frequencies of all DC subsets were observed on day 7 for *lncIrf8* promoter KO cells compared to control (Figure 3A–F). In spleen, frequencies of cell populations from *lncIrf8* promoter KO and control were similar to bone marrow, including lower frequencies of all DC subsets for *lncIrf8* promoter KO (Figure 3G–L). CD45.2 donor HoxB8 cells were largely lost at day 14 after cell transplantation (Figure 3B–F and Figure 3H–L).

**Figure 3—figure supplement 1.**
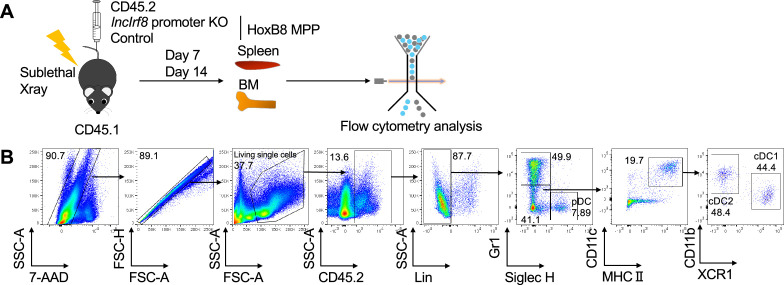
Cell transplantation of HoxB8 MPP. (**A**) Workflow of HoxB8 MPP transplantation. CD45.1 recipient mice were sublethal irradiated and injected with CD45.2 *lncIrf8* promoter KO and control HoxB8 MPP. Seven and 14 days after cell transplantation, mice were sacrificed and cells from bone marrow and spleen were isolated and subjected to flow cytometry analysis for DC subsets. (**B**) Gating strategy for CD45.2 DC subsets following transplantation of CD45.2 *lncIrf8* promoter KO HoxB8 MPP into CD45.1 recipient mice. 7-AAD^-^ CD45.2^+^ lineage^-^ donor cells were gated on Gr1^+^ monocytes, Gr1^-^ Siglec H^+^ pDC, and Gr1^-^Siglec H^-^ CD11c^+^ MHCII^+^ DC, and further on cDC1 and cDC2 subsets. cDC1 are Gr1^-^ Siglec H^-^ MHCII^+^ CD11c^+^ CD11b^low/-^ XCR1^+^ and cDC2 are Gr1^-^ Siglec H^-^ MHCII^+^ CD11c^+^ CD11b^+^ XCR1^-^. The lineage cocktail (Lin) was: Ter119, CD19, CD3e, NK1.1, F4/80.

Thus, *lncIrf8* promoter KO compromised pDC and cDC1 development both in vitro and in vivo.

### *lncIrf8* acts as an indicator of *Irf8* +32 kb enhancer activity in pDC

**Figure 4.**
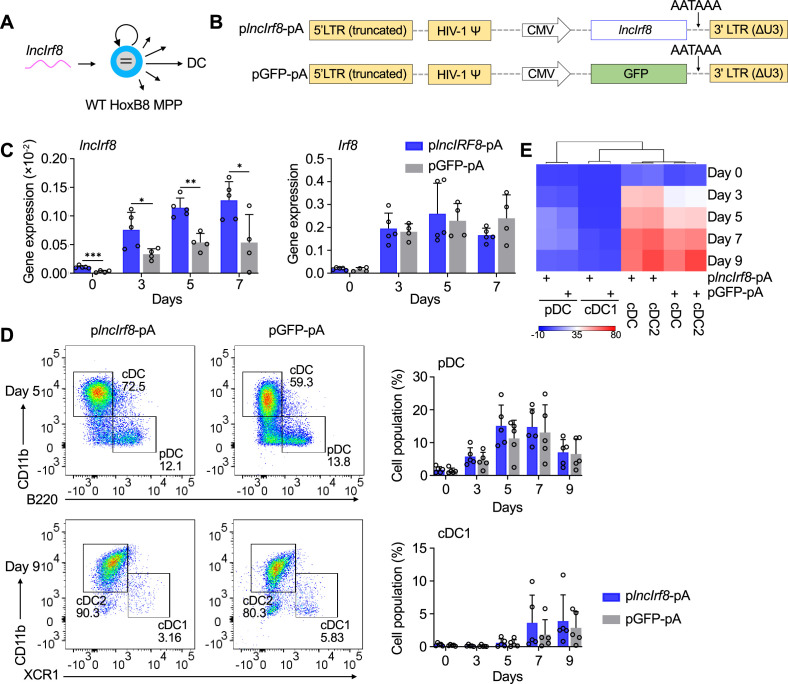
*lncIrf8* overexpression leaves pDC and cDC1 development unaffected. (**A and B**) Schematic representation of *lncIrf8* overexpression in WT HoxB8 MPP and of p*lncIrf8*-pA (*lncIrf8* overexpression) and pGFP-pA (control) plasmids. A polyA signal AATAAA for transcription termination was inserted at the 3’ end of *lncIrf8* and GFP. (**C**) Gene expression of *lncIrf8* and *Irf8* in p*lncIrf8*-pA and pGFP-pA HoxB8 MPP on day 0, 3, 5, and 7 of Flt3L directed DC differentiation (n=4–5). Gene expression was by RT-qPCR and normalized to *GAPDH*. (**D**) Representative flow cytometry of DC subsets at day 5 and 9 of Flt3L directed DC differentiation of p*lncIrf8*-pA and pGFP-pA HoxB8 MPP. Quantification of pDC and cDC1 of living single cells on Flt3L directed DC differentiation day 0, 3, 5, 7, and 9 (n=5) is shown. Gating for pDC and cDC1 was as in Figure 2—figure supplement 1E. (**E**) Heatmap representation of DC subsets of panel (**D**) at day 0, 3, 5, 7, and 9 of DC differentiation. Red, high frequency; white, intermediate frequency and blue, low frequency. Data represent mean ± SD of at least three independent experiments with different HoxB8 MPP clones of p*lncIrf8*-pA and pGFP-pA. *p<0.05, **p<0.01, ***p<0.001, multiple t-tests. Data that have no difference (p>0.05) are not labeled.

Knockout of *lncIrf8* promoter and thus abolishment of *lncIrf8* expression severely diminished pDC and cDC1 development in vitro and in vivo. The *lncIrf8* promoter is located within *Irf8* +32 kb enhancer (Durai et al., 2019) and thus it was important to determine whether *lncIrf8* itself plays a role in regulating pDC and cDC1 development. To address this question, we (i) overexpressed *lncIrf8* in wild-type MPP and (ii) re-expressed *lncIrf8* in *lncIrf8* promoter KO MPP and monitored its impact on DC development (Figure 4 and Figure 4—figure supplement 1).

**Figure 4—figure supplement 1.**
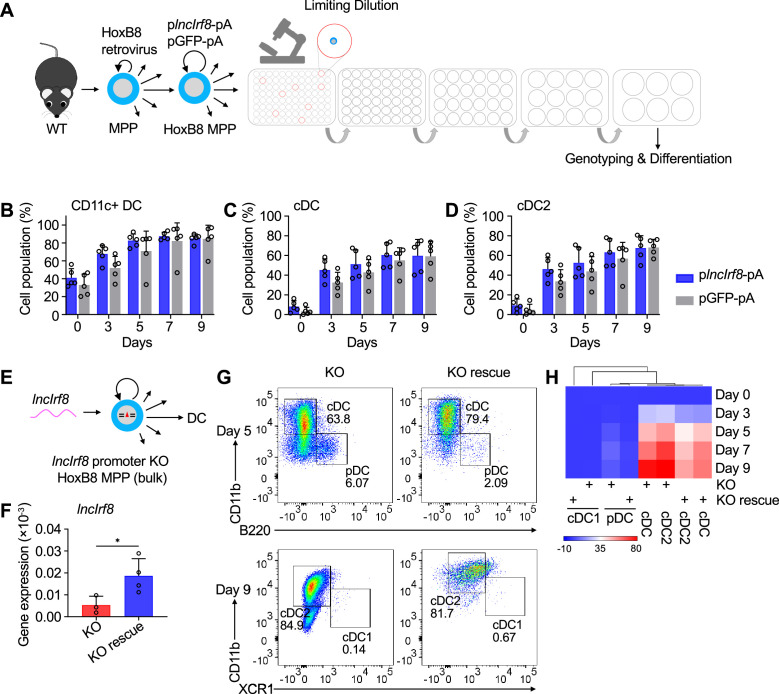
*lncIrf8* overexpression and rescue left DC differentiation unaffected. (**A**) WT HoxB8 MPP were infected with p*lncIrf8*-pA (*lncIrf8* overexpression) and pGFP-pA (control) lentiviral particles, followed by limiting dilution to obtain single cell colonies. HoxB8 MPP clones were screened by genomic PCR and the positive colonies were subjected to DC differentiation. (**B–D**) Quantification of CD11c^+^ DC, all cDC and cDC2 of living single cells in p*lncIrf8*-pA and pGFP-pA HoxB8 MPP on day 0, 3, 5, 7, and 9 of Flt3L directed DC differentiation (n=5). Gating was as in Figure 2—figure supplement 1E. Data represent mean ± SD of at least three independent experiments with different HoxB8 MPP clones of p*lncIrf8*-pA and pGFP-pA. Data were calculated by multiple t-tests and data that have no difference (p>0.05) are not labeled. (**E**) Schematic representation of *lncIrf8* rescue in *lncIrf8* promoter KO HoxB8 MPP (bulk). (**F**) Gene expression of *lncIrf8* in *lncIrf8* promoter KO and KO rescue HoxB8 MPP. Gene expression was by RT-qPCR and normalized to *GAPDH*. Data represent mean ± SD of at least three independent experiments and were calculated by unpaired t test. *p<0.05. (**G**) Representative flow cytometry analysis of DC subsets pDC, cDC1 and cDC2 at day 5 and 9 of Flt3L directed DC differentiation of *lncIrf8* promoter KO rescue and control (KO). (**H**) DC subsets pDC, all cDC, cDC1 and cDC2 were calculated in percent of living single cells in *lncIrf8* promoter KO and KO rescue on day 0, 3, 5, 7, and 9 of Flt3L directed DC differentiation and data are represented in heat map format; n=3–4 from at least three independent experiments. Red, high frequency; white, intermediate frequency and blue, low frequency.

*lncIrf8* cDNA was cloned into a polyA lentivirus vector. An ‘AATAAA’ stop signal (Alvarez-Dominguez et al., 2015) was inserted at the 3’ end of *lncIrf8* to avoid longer transcripts than *lncIrf8* (p*lncIrf8*-pA, Figure 4B). The respective pGFP-pA vector was used as control. *lncIrf8* overexpressing single-cell clones were generated by limiting dilution (Figure 4—figure supplement 1A), expanded and subjected to DC differentiation. As expected *lncIrf8* expression was markedly increased in p*lncIrf8*-pA infected cells compared to control, while there were no significant differences in *Irf8* expression between the two groups during DC differentiation (Figure 4C). Further, there were no differences in the frequencies of pDC, cDC1, and cDC2 between p*lncIrf8*-pA infected cells and controls (Figure 4D and E, Figure 4—figure supplement 1B–D), indicating that *lncIrf8* overexpression has no effect on *Irf8* expression and DC differentiation.

To further extend this observation we performed a *lncIrf8* rescue in *lncIrf8* promoter KO MPP. *lncIrf8* was re-expressed in *lncIrf8* promoter KO MPP by lentiviral vector and cells were subjected to DC differentiation (Figure 4—figure supplement 1E). *lncIrf8* RNA was effectively expressed and cells differentiated in response to Flt3L (Figure 4—figure supplement 1F). Yet frequencies of pDC and cDC1 were very low to absent and not rescued by *lncIrf8* expression (Figure 4—figure supplement 1G, H).

In a nutshell, *lncIrf8* overexpression and rescue had no effects on pDC and cDC1 development. This strongly suggests that *lncIrf8* has no activity on its own in DC differentiation but rather acts as an indicator for the activation state of sequences within the *Irf8* +32 kb enhancer. In addition, the *lncIrf8* promoter comprises a sequence element with impact on pDC and cDC1 development.

### Activation of *lncIrf8* promoter promotes cDC1 development

**Figure 5.**
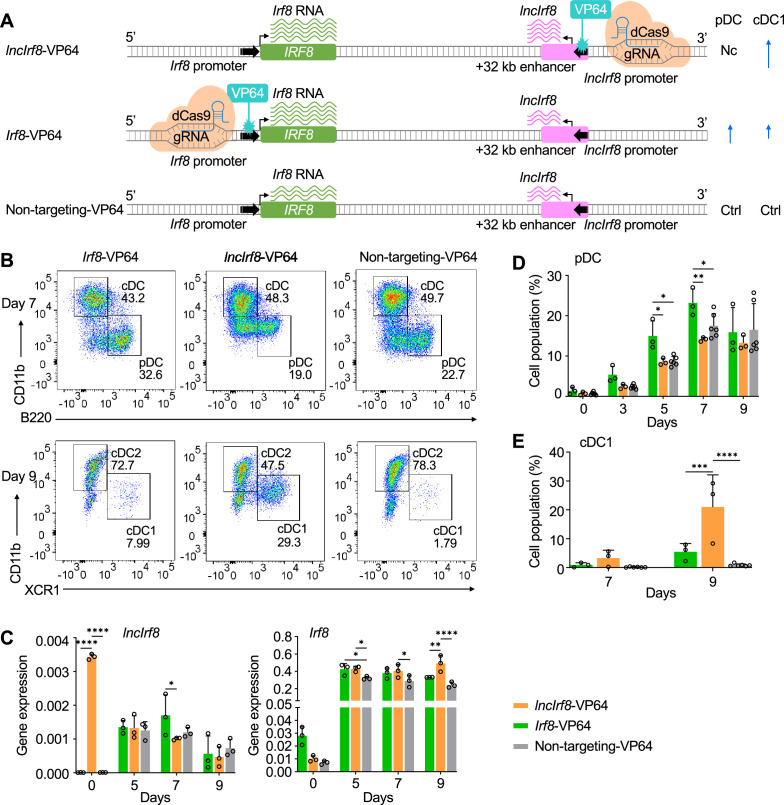
Activation of *lncIrf8* promoter promotes cDC1 development. (**A**) Schematic representation of *lncIrf8* and *Irf8* promoter activation (top and middle, respectively) by CRISPR activation with dCas9-VP64. gRNAs were positioned upstream of *lncIrf8* and *Irf8* TSS for gene activation. dCas9-VP64 cells with non-targeting gRNA were used as control (bottom). Green and purple wavy lines represent *Irf8* and *lncIrf8* RNA, respectively. The number of wavy lines indicates levels of gene transcription determined by RT-qPCR in (**C**). Different length of blue arrows represents the frequencies of pDC and cDC1 according to panel B, D and E. Nc, No change; Ctrl, Control. (**B**) Representative flow cytometry analysis of CRISPR activation targeting the *lncIrf8* and *Irf8* promoters at day 7 and 9 of Flt3L directed DC differentiation. Two non-targeting gRNAs were used as controls and one representative non-targeting gRNA is shown (Non-targeting-VP64). Top row, CD11b^+^ B220^-^ cDC and CD11b^-^ B220^+^ pDC at day 7 of Flt3L directed DC differentiation; bottom row, CD11b^low/-^ XCR1^+^ cDC1 and CD11b^+^ XCR1^-^ cDC2 at day 9 of DC differentiation. For gating strategy see Figure 5—figure supplement 1B. (**C**) Gene expression of *lncIrf8* and *Irf8* in *lncIrf8*-VP64, *Irf8*-VP64 and non-targeting-VP64 HoxB8 MPP on day 0, 5, 7, and 9 of Flt3L directed DC differentiation (n=3). Gene expression analysis was by RT-qPCR and data are normalized to *GAPDH*. Data represent mean ± SD of three independent experiments. *p<0.05, **p<0.01, ****p<0.0001, two-way ANOVA, Tukey’s multiple comparisons test. Data that have no difference (p>0.05) are not labeled. (**D and E**) Quantification of pDC and cDC1 in percent of living single cells as in panel (**B**) on various days of Flt3L directed DC differentiation (n=3). Non-targeting-VP64 refers to both non-targeting-VP64 controls (n=6). Data represent mean ± SD of 3 independent experiments. *p<0.05, **p<0.01, ***p<0.001, ****p<0.0001, two-way ANOVA, Tukey’s multiple comparisons test. Data that have no difference (p>0.05) are not labeled.

Next, we proceeded to study the impact of the sequence element within *lncIrf8* promoter on *Irf8* expression and DC differentiation using CRISPR activation by dCas9-VP64 (Figure 5A and Figure 5—figure supplement 1). dCas9-VP64 is a mutated Cas9 deficient in nuclease activity, which is fused to the VP64 effector domain and confers gene activation. Targeting of dCas9-VP64 to the *lncIrf8* promoter was achieved with specific gRNAs (Figure 5—figure supplement 1I). We also included targeting dCas9-VP64 to the *Irf8* promoter to study the interplay with the *lncIrf8* promoter. FACS sorted HoxB8 MPP expressing dCas9-VP64 and gRNA were subjected to DC differentiation and analyzed for *lncIrf8* and *Irf8* expression and DC subset composition (Figure 5B–E and Figure 5—figure supplement 1A–E).

**Figure 5—figure supplement 1.**
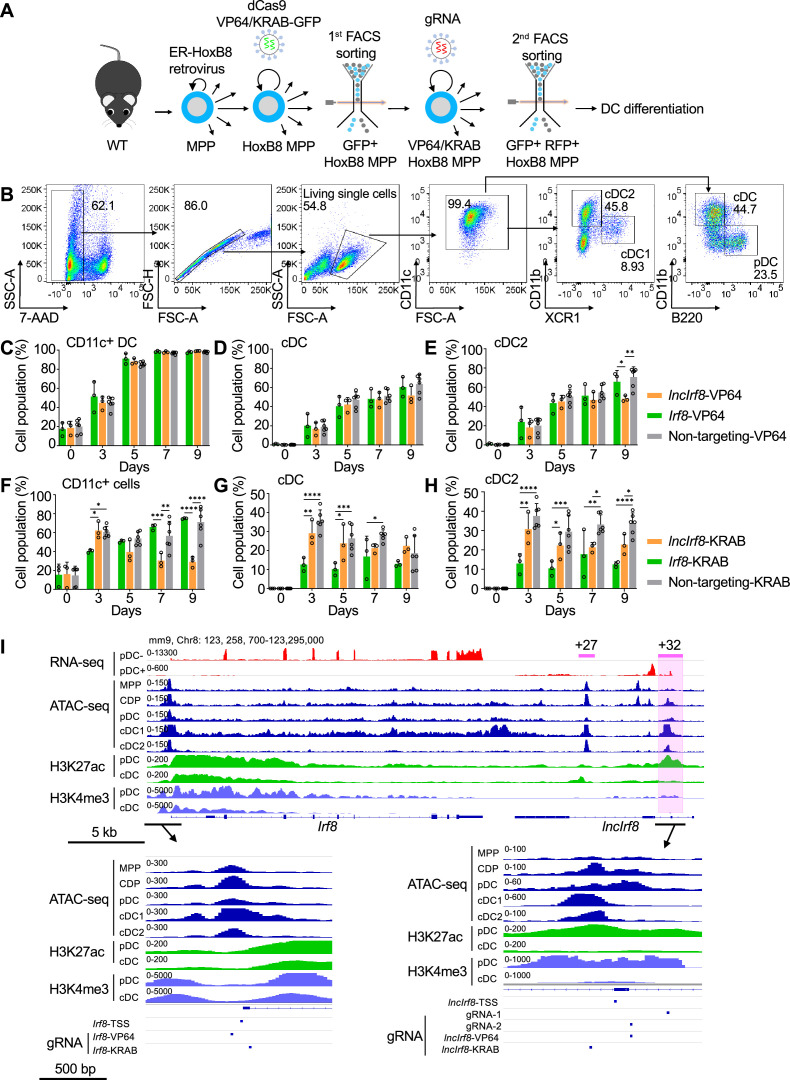
CRISPR/dCas9 mediated gene activation and repression, and gRNA positions. (**A**) WT HoxB8 MPP were infected with dCas9-VP64-GFP and KRAB-dCas9-GFP lentiviral vectors followed by 1^st^ FACS sorting for GFP^+^ cells to obtain dCas9-VP64-GFP and dCas9-KRAB-GFP HoxB8 MPP. Gene targeting of dCas9-VP64 and dCas9-KRAB was achieved by infection of MPP with specific gRNA vectors and 2^nd^ FACS sorting for GFP^+^ RFP^+^ cells. (**B**) Gating strategy for DC subsets in CRISPR/dCas9 HoxB8 MPP upon DC differentiation. DC subsets were gated from 7-AAD^-^ CD11c^+^ cells, and further gated on all cDC, cDC1, cDC2 and pDC as in Figure 2—figure supplement 1E. (**C–H**) Quantification of CD11c^+^ cells, all cDC, and cDC2 of panel (**B**) in percent of living single cells in dCas9-VP64 cells and dCas9-KRAB cells on day 0, 3, 5, 7, and 9 of Flt3L directed DC differentiation (n=3). Non-targeting-VP64 and Non-targeting-KRAB refers to non-targeting-VP64 and KRAB controls, respectively (n=6). Data represent mean ± SD of 3 independent experiments. *P<0.05, **P<0.01, ***P<0.001, ****P<0.0001, two-way ANOVA, Tukey’s multiple comparisons test. Data that have no difference (*P*>0.05) are not labeled. (**I**) Gene expression and epigenetic landscape of *Irf8* gene and its flanking regions in MPP, CDP, pDC, all cDC, cDC1 and cDC2 are depicted as in Figure 1A and Figure 1—figure supplement 1. *Irf8* +27 kb and +32 kb enhancer (purple line and box, respectively), transcription start sites of *Irf8* (*Irf8*-TSS) and *lncIrf8* (*lncIrf8*-TSS) are indicated. Bottom, positions of gRNAs used in this study: gRNA1 and gRNA2, *lncIrf8* promoter KO by the CRISPR/Cas9 system; *Irf8*-VP64 and KRAB gRNAs for activation and repression of *Irf8* promoter, respectively; *lncIrf8*-VP64 and KRAB gRNAs for activation and repression of *lncIrf8* promoter, respectively.

Activation of the *lncIrf8* promoter by dCas9-VP64 caused a massive increase of *lncIrf8* expression at DC differentiation day 0 (Figure 5C). The activation of the *lncIrf8* promoter led also to *Irf8* upregulations at DC differentiation day 5, 7, and 9 compared to non-targeting control (Figure 5A and C). This demonstrates the positive impact of the *lncIrf8* promoter element on *Irf8* expression during DC differentiation and is in accord with the physical interaction of *lncIrf8* and *Irf8* promoters by Capture-C (Figure 1B and Figure 1—figure supplement 1).

Intriguingly, in *lncIrf8*-VP64 cells *lncIrf8* expression was downregulated at DC differentiation day 5, 7 and 9, while *Irf8* expression was upregulated (Figure 5A and C). This indicates a repressive effect of IRF8 on *lncIrf8* promoter and is in accord with IRF8 binding to the *lncIrf8* region by ChIP-seq (Figure 1 and Figure 1—figure supplement 1). This observation suggests a negative feedback loop of IRF8 on the *lncIrf8* promoter during DC differentiation (Figure 5A and C).

Activation of *Irf8* promoter by dCas9-VP64 increased *Irf8* expression at DC differentiation day 0, 5, 7, and 9, while expression of *lncIrf8* was unaffected compared to non-targeting control (Figure 5A and C). As expected *Irf8* promoter activation led to higher pDC frequencies (Figure 5B and D), and also increased cDC1 frequencies compared to the non-targeting controls (Figure 5B and E). Importantly, *lncIrf8* promoter activation by dCas9-VP64 also increased cDC1 frequencies and this was particular prominent at day 9 of DC differentiation (Figure 5B and E). Frequencies of cDC2 were decreased at day 9 and other populations, including pDC, remained unchanged (Figure 5B and D, Figure 5—figure supplement 1C–E).

Taken together our CRISPR activation of the *lncIrf8* and *Irf8* promoters by dCas9-VP64 suggest a negative feedback loop of *Irf8* for pDC and cDC1 development.

### Negative feedback regulation of *lncIrf8* and *Irf8* promoters controls DC differentiation

**Figure 6.**
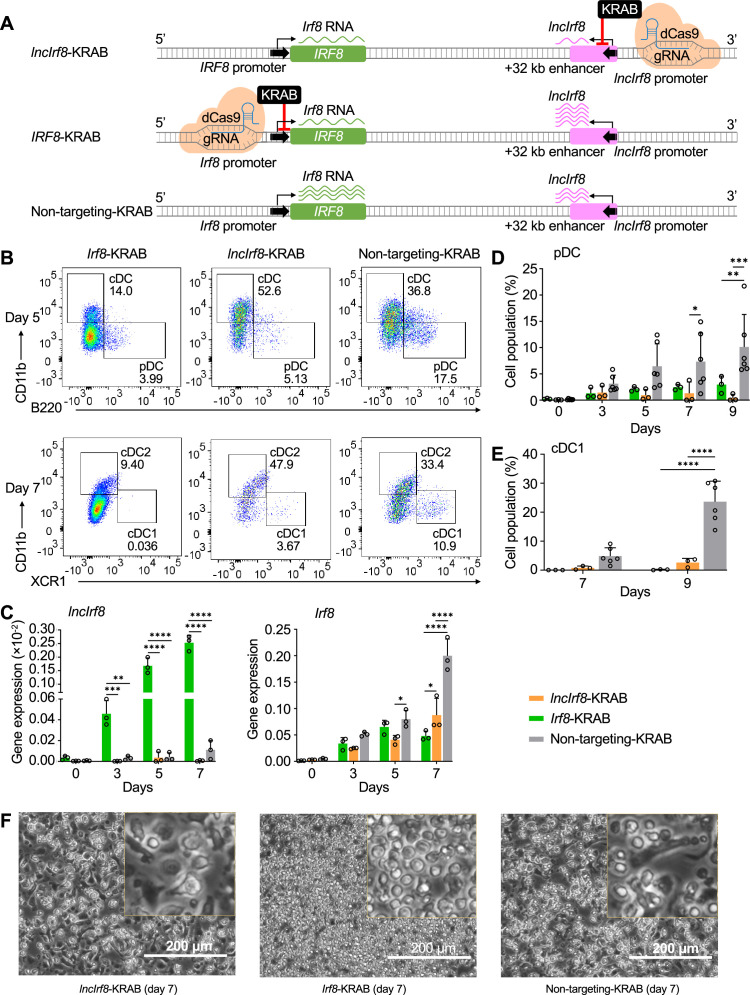
Repression of *lncIrf8* promoter compromises pDC and cDC1 development. (**A**) Schematic representation of *lncIrf8* and *Irf8* promoter repression (top and middle, respectively) by CRISPR interference with dCas9-KRAB. gRNAs were positioned downstream of *lncIrf8* and *Irf8* TSS to block gene transcription. dCas9-KRAB cells with non-targeting gRNA were used as control (bottom). Green and purple wavy lines represent *Irf8* and *lncIrf8* RNA, respectively. The number of wavy lines indicates levels of gene transcription determined by RT-qPCR in (**C**). (**B**) Representative flow cytometry analysis of CRISPR interference targeting the *lncIrf8* and *Irf8* promoters at day 5 and 7 of Flt3L directed DC differentiation. Two non-targeting gRNAs were used as controls and one representative non-targeting gRNA is shown (Non-targeting-KRAB). cDC and pDC at day 5 and cDC1 and cDC2 at day 7 of Flt3L directed DC differentiation are shown similar to Figure 5B. (**C**) Gene expression of *lncIrf8* and *Irf8* in *lncIrf8*-KRAB, *Irf8*-KRAB and non-targeting-KRAB HoxB8 MPP on day 0, 3, 5, and 7 of Flt3L directed DC differentiation (n=3). Gene expression analysis was by RT-qPCR and data are normalized to *GAPDH*. Data represent mean ± SD of three independent experiments. *p<0.05, **p<0.01, ***p<0.001, ****p<0.0001, two-way ANOVA, Tukey’s multiple comparisons test. Data that have no difference (p>0.05) are not labeled. (**D and E**) Quantification of pDC and cDC1 in percent of living single cells as in panel (**B**) on various days of Flt3L directed DC differentiation (n=3). Non-targeting-KRAB refers to both non-targeting-KRAB controls (n=6). Data represent mean ± SD of three independent experiments. *p<0.05, **p<0.01, ***p<0.001, ****p<0.0001, two-way ANOVA, Tukey’s multiple comparisons test. Data that have no difference (p>0.05) are not labeled. (**F**) Representative phase-contrast microscopy image of *lncIrf8*-KRAB, *Irf8*-KRAB and non-targeting-KRAB on day 7 of Flt3L directed DC differentiation. Scale bar: 200 μm.

To directly investigate the negative feedback loop of *Irf8* regulation, we repressed *lncIrf8* and *Irf8* promoters by targeted repression with dCas9-KRAB and analyzed the DC subsets during DC differentiation (Figure 6A–F and Figure 5—figure supplement 1A, B, F-H). dCas9-KRAB is a nuclease deficient Cas9 fused to the KRAB effector domain, which confers gene repression when positioned with specific gRNA (Figure 5—figure supplement 1I).

Targeted repression of the *Irf8* promoter decreased *Irf8* expression as expected but massively increased *lncIrf8* expression compared to non-targeting control (Figure 6A and C). This is very much in line with *Irf8* impacting *lncIrf8* expression by a negative feedback loop. Positioning the dCas9-KRAB repressor in the Inc*IRF8* promoter led to downregulation of both *Irf8* and *lncIrf8* expression, which confirms the *lncIrf8* promoter element acting on the *Irf8* promoter (Figure 6A and C).

Interestingly, targeted repression of the *Irf8* promoter severely compromised development of all DC subsets, including pDC, cDC1, and cDC2, compared to non-targeting controls and yielded CD11c^+^ cells with progenitor-like spherical morphology (Figure 6B and D–F, Figure 5—figure supplement 1F–H). This reemphasizes the pivotal role of IRF8 for DC development known from studies on *Irf8* knockout mice (Murakami et al., 2021; Schiavoni et al., 2002; Sichien et al., 2016; Tsujimura et al., 2003). In addition, targeted repression of the *lncIrf8* promoter (*lncIrf8*-KRAB) also compromised pDC and cDC1 development (Figure 6B, D and E). This result is very similar to the *lncIrf8* promoter KO analyzed above (Figure 2A–C).

**Figure 7.**
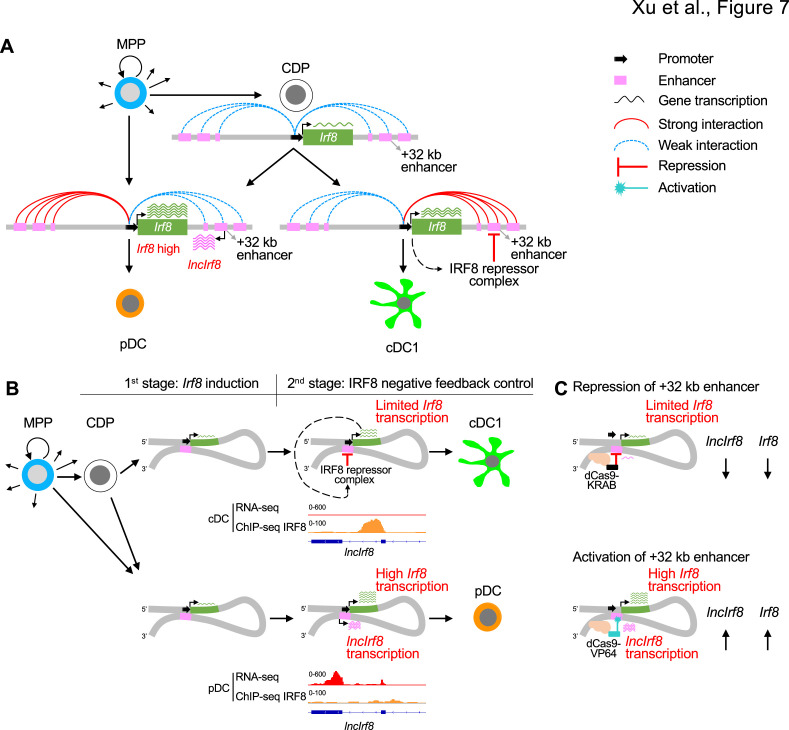
Negative feedback loop of *Irf8* through +32 kb enhancer governs DC differentiation. (**A**) Schematic representation of *Irf8* gene regulation during DC differentiation. *Irf8* transcription is induced at the CDP stage by its flanking enhancers, including the +32 kb enhancer, and is further increased in pDC and cDC1 (green wavy lines; the number of wavy lines indicates levels of gene transcription as in Figure 5A and Figure 6A). The increase in *Irf8* expression is due to an increase in the interactions of the *Irf8* promoter with upstream and downstream sequences. *Irf8* promoter interactions with upstream sequences are stronger in pDC and *Irf8* promoter interactions with downstream sequences are stronger in cDC1. In pDC the +32 kb enhancer marked by *lncIrf8* is less repressed by IRF8 repressor complex compared to cDC1, resulting in particularly high *Irf8* expression and *lncIrf8* transcription in pDC (purple wavy lines). This negative feedback inhibition of IRF8 on the +32 kb enhancer allows *Irf8* to regulate its own expression and thus DC differentiation. (**B**) Negative feedback regulation of *Irf8* through the +32 kb enhancer in cDC1 and pDC specification as described in panel (**A**). The RNA-seq and IRF8 ChIP-seq data are shown as in Figure 1A, Figure 1—figure supplement 1 and Figure 2—figure supplement 2A. (**C**) Recapitulating *Irf8* and *lncIrf8* transcription by repression and activation of the *Irf8* +32 kb enhancer in HoxB8 MPP by targeted dCas9-KRAB and dCas9-VP64, respectively. Green and purple wavy lines represent gene expression as described in Figure 5A and Figure 6A.

All these findings support a model of *Irf8* regulating its own expression by a negative feedback loop acting on the *Irf8* +32 kb enhancer to limit *Irf8* autoactivation (Figure 7). This regulatory *Irf8* +32 kb enhancer element is marked by *lncIrf8. Irf8* expression starts in CDP and further increases in pDC and cDC1, with particularly high expression in pDC (Figure 1A, Figure 7A and B, Figure 1—figure supplement 1). The increase in *Irf8* expression is proposed to be due to an increase in the interactions of the *Irf8* promoter with upstream and downstream sequences. In pDC, the *Irf8* promoter-enhancer interactions are more with upstream chromatin regions (Figure 1C and Figure 7A), which relates to high *Irf8* expression. In cDC1, the *Irf8* promoter interactions are stronger with the regions downstream of *Irf8* (Figure 1C and Figure 7A) and the *Irf8* +32 kb enhancer marked by *lncIrf8* confers transcriptional repression.

In our model, we propose an IRF8 repressor complex that differentially acts on the *Irf8* +32 kb enhancer element in a DC subset specific manner to limit *Irf8* autoactivation. In cDC1 the +32 kb enhancer is repressed by the IRF8 repressor complex through negative feedback inhibition (prominent IRF8 binding in cDC by ChIP-seq; Figure 7A and B), which limits *Irf8* autoactivation and expression. Conversely, in pDC there is less IRF8 repressor complex binding to the +32 kb enhancer, which results in high *Irf8* and *lncIrf8* transcription (Figure 7A and B). Recapitulating the IRF8 repressor complex with dCas9-KRAB and targeting *lncIrf8* promoter in the +32 kb enhancer reduced *Irf8* expression (Figure 7C). Conversely, activation of the +32 kb enhancer boosted *lncIrf8* and *Irf8* expression (Figure 7C). Thus, an intricate feedback loop of IRF8 on the +32 kb enhancer orchestrates *Irf8* expression and thus DC differentiation.

## Discussion

Hematopoiesis is a particularly well studied stem cell system and therefore provides an excellent model for studying TF in lineage commitment and cell differentiation, and the molecular principles involved (Belz and Nutt, 2012; Graf and Enver, 2009; Laurenti and Göttgens, 2018; Nutt and Chopin, 2020). This work revealed a previously unrecognized negative feedback loop of *Irf8* in DC differentiation and shows how *Irf8* autoactivation is controlled and ultimately limited. IRF8 is crucial for DC lineage specification both in humans and mice (Anderson et al., 2021; Belz and Nutt, 2012; Cabeza-Cabrerizo et al., 2021; Cytlak et al., 2020; Kurotaki et al., 2019; Nutt and Chopin, 2020). *Irf8* is upregulated by autoactivation via the +32 kb enhancer (Grajales-Reyes et al., 2015; Lin et al., 2015). However, how *Irf8* expression is controlled at late stages of DC differentiation and eventually limited is not known. Here we demonstrate that *Irf8* expression is limited by a negative feedback loop via a sequence element marked by *lncIrf8* in the *Irf8* +32 kb enhancer.

*Irf8* expression in hematopoiesis is regulated by its flanking enhancers, which determine lineage specification and DC subset development (Bagadia et al., 2019; Durai et al., 2019; Grajales-Reyes et al., 2015; Murakami et al., 2021; Schönheit et al., 2013). Frequently, enhancers are identified by ATAC-seq and ChIP-seq (Durai et al., 2019; Murakami et al., 2021), and here we embarked on a different approach and searched for eRNA and enhancer-associated lncRNA by RNA-seq. We identified a novel pDC-specific lncRNA (*lncIrf8*) transcribed from the *Irf8* +32 kb enhancer. *lncIrf8* itself lacks activities in DC differentiation but a *lncIrf8* promoter element is crucial for pDC and cDC1 development. Upon deletion of this *lncIrf8* promoter element pDC and cDC1 development was severely compromised, demonstrating that this sequence is important for both pDC and cDC1 development.

We propose a model where *Irf8* expression during DC differentiation is in a first step initiated and activated through flanking enhancers, including the +32 kb enhancer by autoactivation (Grajales-Reyes et al., 2015; Lin et al., 2015; Figure 7). In a second step, the *lncIrf8* promoter element confers feedback inhibition, which limits *Irf8* expression. This feedback inhibition is different for pDC and cDC1, both of which express high levels of *Irf8* (Bornstein et al., 2014; Grajales-Reyes et al., 2015; Lin et al., 2015). In cDC1, *Irf8* expression is attributed to *Irf8* autoactivation through the +32 kb enhancer driven by the BATF3-IRF8 complex (Durai et al., 2019; Grajales-Reyes et al., 2015). In pDC, *Irf8* expression is even higher than in cDC1 (Bornstein et al., 2014; Lin et al., 2015), which we propose is due to less feedback inhibition at late stages of DC differentiation.

A candidate for mediating *Irf8* feedback inhibition is IRF8 itself, since IRF8 works as transcriptional activator or repressor, depending on context. IRF8 activator or repressor function depends largely on the heterodimers (or even heterotrimers) with partner TF that bind to specific DNA sequences (Chang et al., 2018; Huang et al., 2016; Humblin et al., 2017; Tamura et al., 2015; Yoon et al., 2014). Modifications on IRF8 protein, such as phosphorylation and small molecule conjugation, also alter IRF8 activity (Chang et al., 2012; Konieczna et al., 2008). Potential IRF8 heterodimer partners, to form repressor complexes, are ETV6 or IRF2 (Huang et al., 2016; Humblin et al., 2017; Lau et al., 2018). The IRF8 repressor complex is proposed to bind to the +32 kb enhancer in cDC but not in pDC. This notion is in line with a prominent IRF8 signal at the +32 kb enhancer in cDC, which is absent in pDC (Durai et al., 2019; Grajales-Reyes et al., 2015; Figure 1A, Figure 7A and B, Figure 1—figure supplement 1 and Figure 2—figure supplement 2A).

Further support of our model stems from our CRIPSR activation/interference experiments (Figure 7C). CRIPSR activation of *Irf8* promoter by dCas9-VP64 mimics *Irf8* up-regulation during DC differentiation and causes an increase in pDC and cDC1 (Figure 5). CRIPSR interference of *lncIrf8* promoter by dCas9-KRAB recapitulates transcriptional repressor binding to the +32 kb enhancer and causes *Irf8* promoter inhibition (Figure 6).

We extended our study to delineate the chromatin conformation of the *Irf8* promoter with flanking sequences by Capture-C. The *Irf8* promoter was found to interact with its flanking enhancers already at the CDP stage and then interactions with specific upstream and downstream sequences are proposed to guide pDC and cDC specification, respectively. This is in accord with previous studies where some of these *Irf8* flanking enhancers were required to maintain high levels of *Irf8* expression (Anderson et al., 2021; Murakami et al., 2021).

We demonstrate that deletion of the *lncIrf8* promoter element severely decreased *Irf8* expression and abolished both pDC and cDC1 development in vitro and in vivo upon cell transplantation. These results are very similar to a previous study on cDC1 specific +32 kb enhancer knockout mice, which demonstrates the impact of +32 kb enhancer for cDC1 development in vivo (Durai et al., 2019). The *lncIrf8* promoter is located in close proximity to the cDC1 specific +32 kb enhancer and thus can be expected to have overlapping functions. Indeed, deletion of the cDC1 +32 kb enhancer in HoxB8 MPP showed some similar activities on DC differentiation as the deletion of the *lncIrf8* promoter element, including regulation of *lncIrf8* and *Irf8* expression, but mainly affected cDC1 differentiation. These observations indicate that the *lncIrf8* promoter element has further functions compared to the cDC1 specific +32 kb enhancer, for example for pDC development, and the underlying mechanisms warrant further investigation.

eRNA and enhancer-associated lncRNA are indicative of enhancer activity, however whether the process of their transcription, the transcripts themselves, or both are functionally linked to enhancer activity, remains unclear (Sartorelli and Lauberth, 2020; Statello et al., 2021). Previous studies revealed that some eRNA and enhancer-associated lncRNA are indeed functionally connected with expression of the respective target genes (Sartorelli and Lauberth, 2020; Statello et al., 2021). Here, we found no apparent activity of *lncIrf8* on its own in pDC and cDC1 development, as demonstrated by *lncIrf8* overexpression and rescue experiments. *lncIrf8* appears to rather serve as an indicator for *Irf8* +32 kb enhancer activity. However, *lncIrf8* might have additional functions in DC biology, which are not revealed in the current study and remain to be identified.

In conclusion, by analyzing the gene expression and epigenetic profiles of the *Irf8* locus, we identified an enhancer element marked by *lncIrf8* that regulates *Irf8* and controls DC differentiation through a negative feedback loop. Our results suggest that *Irf8* regulates itself by its flanking enhancers in DC fate determination: First, *Irf8* induces its expression by autoactivation via flanking enhancers, including the *Irf8* +32 kb enhancer, to initiate DC differentiation, and second limits its expression at late stages via the *lncIrf8* promoter element within the same +32 kb enhancer by feedback inhibition. This molecular principle of feedback inhibition is expected to also apply to other TF and cell differentiation systems.

## Materials and methods

### Mice

Wild type C57BL/6, Mx-Cas9-GFP knock-in mice (Kühn et al., 1995; Platt et al., 2014; Xu et al., 2022), and CD45.1 recipient C57BL/6 mice were used in this study. Mice were kept under specific pathogen-free conditions in the central animal facility of RWTH Aachen University Hospital, Aachen, Germany. All the animal experiments were approved by the local authorities of the German State North Rhine-Westphalia, Germany according to the German animal protection law (reference number: 81–02.04.2018 .A228).

### Cell culture

Multipotent progenitors (MPP) were obtained from mouse bone marrow and expanded in vitro with a two-step protocol as described in Felker et al., 2010. Conditionally immortalized HoxB8 MPP were generated by retrovirus infection of bone marrow cells from wild-type or Mx-Cas9-GFP knock-in mice with an estrogen (E2) inducible HoxB8 estrogen receptor (ER) fusion gene (HoxB8-ER) (Redecke et al., 2013; Xu et al., 2022). MPP were grown in RPMI 1640 medium with 10% FCS (Gibco, 10270106), 100 U/ml penicillin/streptomycin, 2 mM L-glutamine and 50 μM β-ME with a four-cytokine cocktail of SCF, Flt3 ligand (Flt3L), IGF-1, and IL-6/soluble IL-6 receptor fusion protein (hyper-IL-6) as before (referred to as MPP growth medium) (Felker et al., 2010; Lutz et al., 2022; Xu et al., 2022) (Appendix 1-key resources table). E2 (1 µM) was added to activate HoxB8-ER and to maintain the conditionally immortalized state of HoxB8 MPP. Cell density was adjusted to 1.5 million cells/ml every day. HEK293T cells for retrovirus and lentivirus production were grown in DMEM supplemented with 10% FCS (PAA, A01125-499), 100 U/ml penicillin/streptomycin, 2 mM L-glutamine (Appendix 1-key resources table).

### In vitro DC differentiation with HoxB8 MPP

HoxB8 MPP were differentiated into DC using a two-step protocol modified from Felker et al., 2010 and described in Lutz et al., 2022; Xu et al., 2022. In brief, 0.75 million cells/ml were grown in MPP growth medium with 50 ng/ml Flt3L (Peprotech, 300–19) and reduced E2 (0.01 μM) for two days and cell density was kept to 0.75 million cells/ml. To induce DC differentiation, HoxB8 MPP were then washed with PBS to remove cytokines and E2, and cultured in RPMI 1640 medium supplemented with FCS, penicillin/streptomycin, L-glutamine, β-ME (same concentrations as above), and Flt3L (50 ng/ml, Peprotech) (referred to as DC differentiation day 0). Partial medium changes were performed on differentiation day 3 and 6. Spontaneous DC differentiation of HoxB8 MPP was achieved simply by removing E2 from growth medium (SCF, Flt3L, IGF1 and hyper-IL6), and culturing the cells at 1.5 million cells/ml (Lutz et al., 2022; Xu et al., 2022).

### Nuclear-Titrated (NuTi) Capture-C

Wild-type bone marrow cell-derived MPP, CDP, cDC1, cDC2, and pDC were generated in vitro with the two-step protocol as described in Felker et al., 2010. Cell populations were sorted by BD FACSAria IIu or BD FACSMelody, MPP are Gr1^-^ CD117^hi^ CD135^low/-^, CDP are Gr1^-^ CD117^int^ CD135^+^ CD115^+^, cDC1 are CD11c^+^ CD11b^low/-^ XCR1^+^, cDC2 are CD11c^+^ CD11b^+^ XCR1^-^, pDC are CD11c^+^ CD11b^-^ B220^+^.

The chromatin conformation capture (3 C) library preparation protocol used in this study was modified from Downes et al., 2021; Downes et al., 2022 with the reagents listed in Appendix 1-key resources table. MPP, CDP, cDC1, cDC2 and pDC were fixed with formaldehyde (final concentration 2%) and subjected to nuclear isolation according to the protocol in Downes et al., 2022; Li et al., 2019. Nuclei (15–20 million per sample) were digested with DpnII and DNA fragments were ligated by T4 DNA HC ligase. DNA was extracted and purified with Phenol-Chloroform-Isoamyl alcohol (PCI, 25:24:1). DpnII digestion efficiency was determined by SYBR qPCR with the primers listed in Appendix 1-key resources table and the quality of 3 C libraries was investigated by agarose gel (1%) electrophoresis; 3 C samples were used only if the DpnII digestion efficiency was more than 70%.

For *Irf8* promoter viewpoint 2 oligonucleotide probes targeting *Irf8* promoter were designed with the design tool Oligo (https://oligo.readthedocs.io/en/latest/index.html). Oligonucleotide probes are positioned adjacent to the DpnII cut sites on a restriction fragment spanning the *Irf8* promoter (chr8:123,259,948–123,260,530) and 70 bp ssDNA biotinylated oligonucleotides were synthetized by Sigma-Aldrich (listed in Appendix 1-key resources table).

To enrich for fragments containing ligation events with *Irf8* promoter viewpoint, NuTi Capture-C was performed as previously described (Downes et al., 2021; Downes et al., 2022). Briefly, the 3 C libraries prepared from MPP, CDP, cDC1, cDC2 and pDC were sonicated using Covaris S220 to an average size of ~200 bp using standard settings recommended by the manufacturer (time: 180 s, duty cycle: 10%, peak incident power: 175 W, cycles per burst: 200, temperature: 5–9°C). End repair was performed with the NEBNext Ultra II kit (New England Biolabs, E7645S) using 2 µg of sonicated 3 C library in duplicate for each sample. Illumina NEBNext Indices (New England Biolabs, E7500S, and E7335S) were added with a total of 6 cycles of amplification to allow for multiplexing. After this step, duplicates were pooled to increase sample complexity. We enriched samples as per NuTi Capture-C protocol, with two capture rounds in a multiplexed reaction, using 2 µg of each indexed sample as an input for the first capture. The hybridization with biotinylated probes was performed with the KAPA Hyper Capture Reagent Kit (Roche, 9075828001). Each ssDNA biotinylated probe was used at a concentration of 2.9 nM, with a final pool concentration of 5.8 nM, and 4.5 µl of the pooled oligonucleotides were used per sample. Captured DNA was pulled-down with M-270 Streptavidin Dynabeads (Invitrogen, 65305), washed and amplified off the beads with a total of PCR 14 cycles. The DNA obtained after the first capture was used as an input in the second capture round. The experiments were performed for the first and the second biological replicate separately, and then sequenced with NextSeq 550 Illumina System with 300 paired-end or 150 paired-end, respectively.

### NuTi Capture-C data analysis

The Capture-C data was analyzed with CapCruncher (v0.1.1a1) pipeline (https://github.com/sims-lab/CapCruncher; sims-lab, 2022) in capture mode (Downes et al., 2022). The reads were aligned to the mm9 genome assembly with Bowtie2 (Langmead and Salzberg, 2012), with specific options: -p6--very-sensitive. Viewpoint coordinates used were: chr8:123,259,948–123,260,530, 1000 bp around viewpoint was excluded. The data were normalized to ~300 kb region around the viewpoint (chr8:123,132,865–123,433,117).

The Capture-C profiles in Figure 1 and Figure 1—figure supplement 1 show the mean number of unique interactions in two biological replicates, normalized to ~300 kb region around the viewpoint. Differential tracks were created by subtraction of the mean normalized tracks.

### ATAC-seq, ChIP-seq, RNA-seq, and scRNA-seq

ATAC-seq analysis of MPP, CDP, cDC1, cDC2, and pDC was performed by Omni-ATAC-seq (Corces et al., 2017) with minor modifications as described in Li et al., 2019. RNA-seq analysis and ChIP-seq analysis was done as previously described (Allhoff et al., 2016; Lin et al., 2015).

To determin lncRNA expression in mouse ex-vivo pDC, scRNA-seq data of BM and splenic pDC were downloaded from GSE114313 (Rodrigues et al., 2018) and BAM files were converted back into FASTQ files using bamtofastq (https://support.10xgenomics.com/docs/bamtofastq). For visualization of lncRNA expression, we created a custom reference genome of mm9 by following the tutorial: available here. Cellranger pipeline was then processed to generate an expression count matrix of pDC. We next used scanpy (Wolf et al., 2018) to analyze the scRNA-seq data. We filtered cells based on the number of detected genes (<200 or>3500), and the proportion of mitochondrial genes (>10%). After data quality control, we retained 7044 cells for splenic pDC and 8158 cells for BM pDC, respectively. We then log-normalized the count matrix using a scaling factor of 10,000 and selected the top 3000 highly variable genes, which were used for dimensionality reduction based on principle component analysis. The cells were visualized using uniform manifold approximation and projection (UMAP) (Becht et al., 2019).

### Plasmids

psPAX2 (Addgene #12260) and pMD2.G (Addgene #12259) for lentiviral packaging and envelope expressing plasmids were kind gifts from Didier Trono, EPFL, Lausanne, Switzerland. The gRNA expressing vector pLKO5.sgRNA.EFS.tRFP (Addgene #57823) and pLKO5.sgRNA.EFS.tRFP657 (Addgene #57824) were kind gifts from Benjamin Ebert, Harvard Medical School, Boston, USA (Heckl et al., 2014).

For *lncIrf8* overexpression and rescue, *lncIrf8* cDNA was introduced into polyA containing lentivirus vector pGFP-pA to generate p*lncIrf8*-pA. pGFP-pA was constructed from pCIG3 (Addgene #78264; Caviness et al., 2014) by Gibson Assembly (New England Biolabs, E5510S; Gibson et al., 2009). In brief, the WPRE element was removed and a polyA signal ‘AATAAA’ was inserted at the 3’ end of GFP to construct pGFP-pA. *lncIrf8* cDNA was sub-cloned into pGFP-pA using XhoI and SalI with the primers shown in Appendix 1-key resources table to obtain p*lncIrf8*-pA. CRISPR activation and repression of *lncIrf8* and *Irf8* promoters were achieved by dCAS9-VP64_GFP (Addgene #61422) (Konermann et al., 2015) and pTet-KRAB-dCas9-GFP (Xu et al., 2022), respectively.

### Lentivirus infection

gRNAs, *lncIrf8*, dCas9-VP64-GFP, and KRAB-dCas9-GFP were delivered into HoxB8 MPP by lentiviral infection. Briefly, lentiviral particles were produced by calcium phosphate transfection (Graham and van der Eb, 1973) of HEK293T cells with psPAX2, pMD2.G, and the lentiviral transfer vector. At 48 and 72 hours after HEK293T cell transfection, supernatant containing virus particles was collected and concentrated using chondroitin sulfate sodium salt (CSS) and polybrene precipitation (Landazuri et al., 2007; Appendix 1-key resources table). HoxB8 MPP were infected twice with concentrated virus and subjected to Ficoll (Pancoll) purification to remove precipitate and dead cells.

### Genetically modified HoxB8 MPP cell lines

#### *lncIrf8* promoter and cDC1 specific +32 kb enhancer KO:

Mx-Cas9-GFP HoxB8 MPP were used to generate *lncIrf8* promoter and cDC1 specific +32 kb enhancer knockout (KO) HoxB8 MPP by CRISPR/Cas9. Briefly, pairs of gRNAs each for *lncIrf8* promoter KO and cDC1 specific +32 kb enhancer KO (Appendix 1-key resources table) were designed with the IDT online gRNA design tool. For cDC1 specific +32 kb enhancer KO we additionally used the same gRNA sequences from Durai et al., 2019 listed in Appendix 1-key resources table. gRNAs were cloned into pLKO5.sgRNA.EFS.tRFP and pLKO5.sgRNA.EFS.tRFP657 vectors (Heckl et al., 2014) using BsmBI-v2 (Appendix 1-key resources table), respectively.

One 10 cm dish (Bio-One) with 1.8 million HEK293T cells (70%–80% confluency) in 10 ml DMEM plus supplements (see above) was used to produce gRNA expressing lentivirus particles. HEK293T cells were transfected with 10 μg gRNA vector, 7.5 μg psPAX2, and 2.5 μg pMD2.G per gRNA by calcium phosphate transfection and lentivirus particles were harvested 48 and 72 h after transfection.

The gRNA expressing lentiviral particles were used to infect 3 million Mx-Cas9-GFP HoxB8 MPP. Cas9 and GFP expression were induced by mIFNα (1000 IU/ml; Appendix 1-key resources table) for 4 h, followed by FACS sorting for cells expressing the two gRNAs and Cas9 (GFP^+^ RFP^+^ RFP657^+^ cells). Single-cell clones were obtained by single-cell FACS sorting or limiting dilution.

Single-cell clones were genotyped by genomic PCR with primers listed in Appendix 1-key resources table. PCR products were sequenced by Eurofins Genomics and sequences were analyzed by SnapGene. Potential off-targets were routinely predicted by online software tools such as CRISPR-Cas9 gRNA checker (https://eu.idtdna.com/site/order/designtool/in-dex/CRISPR _SEQUENCE). For *lncIrf8* promoter KO, 5 out of 71 single-cell colonies with homozygous deletions were subjected to off-target analysis. The top 2–5 predicted coding or non-coding targets were selected and HoxB8 MPP clones without off-target effects, or where off-target effects occurred in genes that were not expressed in MPP, CDP and DC subsets, were used for further studies (Table 1). For cDC1 specific +32 kb enhancer KO, 5 out of 165 single-cell clones with homozygous deletions were subjected to DC differentiation and further analyzed. Potential off-target genes of self-designed gRNAs for cDC1 +32 kb enhancer KO were not expressed in MPP, CDP, and DC subsets, and thus these gRNAs were used in the study.

**Table 1.** Off-target analysis.

Potential off-targets			KO bulk(100 cells)	KO single-cell clones					Gene expression			
				6	7	19	21	24	MPP	CDP	cDC	pDC
gRNA1	Chr 13: –48032179	*Gm36101*	No	No	No	No	No	No	No	No	No	No
	Chr 2: –131293076	Non-coding	No	No	No	No	No	No	No	No	No	No
gRNA2	Chr 1: –136050990	*AsCl5*	16 bp deletion	16 bp deletion	16 bp deletion	No	1 bp insertion	No	No	No	No	No
	Chr 6:+119352090	*CACNA2D4*	No	No	No	No	No	No	No	No	No	Yes
	Chr 3:+33982811	Non-coding	3–29 bp deletion; 1–3 bp insertion	3–29 bp deletion	3 bp deletion	1–2 bp insertion	No	No	No	No	No	No
	Chr 2: –132060887	Non-coding	No	No	No	No	No	No	No	No	No	No
	Chr 2: –150624255	Non-coding	No	No	No	No	No	No	No	No	No	No

Top potential off-targets of gRNA1 and gRNA2 predicted by CRISPR-Cas9 gRNA checker (see Materials and methods) were analyzed in *lncIrf8* promoter KO bulk culture and KO single-cell clones by PCR analysis of genomic DNA. Potential off-target genes (coding) and non-coding sequences are listed. The absence of off-targets (No) and off-target deletions/insertion are shown.

#### *lncIrf8* overexpression and *lncIrf8* knockout rescue

*lncIrf8* overexpression was performed in wild-type HoxB8 MPP. Lentiviral particles expressing *lncIrf8* were produced from ten 6 cm dishes (Bio-One), each consisting of 0.75 million HEK293T cells (70–80% confluency) in 5 ml DMEM with supplements (see above). HEK293T cells were transfected with 5 μg p*lncIrf8*-pA or pGFP-pA, 2.5 μg psPAX2, and 2.5 μg pMD2.G. Lentivirus particles were concentrated as above and used to infect 0.5 million HoxB8 MPP; single-cell clones were generated by limiting dilution and genotyped using the primers listed in Appendix 1-key resources table. Two out of 47 HoxB8 MPP colonies with p*lncIrf8*-pA and 3 out of 19 HoxB8 MPP colonies with pGFP-pA (control) were expanded and subjected to Flt3L-directed DC differentiation.

*lncIrf8* knockout rescue was carried out in *lncIrf8* promoter KO HoxB8 MPP. FACS sorted cells that genotyped as *lncIrf8* promoter KO homozygous deletion cells, were infected with lentiviral particles expressing *lncIrf8*. Lentiviral infection conditions were the same as for *lncIrf8* overexpression in wild-type HoxB8 MPP (see above).

#### CRISPR activation and CRISPR interference

CRISPR activation and CRISPR interference were performed by infecting wild-type HoxB8 MPP with lentiviral particles expressing dCAS9-VP64_GFP and pTet-KRAB-dCas9-GFP, respectively. The virus particles were produced as in the *lncIrf8* overexpression experiments. In brief, virus particles from ten 6 cm dishes were used to infect 0.5 million wild-type HoxB8 MPP, followed by FACS sorting for GFP^+^ cells expressing dCas9-VP64 or dCas9-KRAB. Doxycycline (1 μg/ml) was used to induce dCas9-KRAB expression 2 days before cell sorting.

gRNAs targeting *lncIrf8* and *Irf8* promoters were cloned into pLKO5.sgRNA.EFS.tRFP as above. The dCas9-VP64-GFP and dCas9-KRAB-GFP HoxB8 MPP were then infected with specific gRNAs for gene activation and repression. The conditions for gRNA infection were the same as in *lncIrf8* promoter KO experiments. Doxycycline (1 μg/ml) was given to the sorted dCas9-KRAB-GFP cells every 3 days to ensure sustained dCas9-KRAB expression.

### Flow cytometry analysis and cell sorting

DC subsets were analyzed by flow cytometry using FACS Canto II or LSR Fortessa (both from BD Biosciences). The information for flow cytometry antibodies is shown in Appendix 1-key resources table. For live/dead staining, cells were incubated with 3 μl 7-AAD per test for 5–10 min before flow cytometry measurement. Cells were sorted by FACS Aria IIu or FACS Melody, and flow cytometry data were analyzed by FlowJo V10 (all from BD Biosciences). Data on DC frequencies were subjected to the hierarchical clustering and represented in heatmap format using the online tool MORPHEUS (https://software.broadinstitute.org/morpheus/).

### Cell transplantation

CD45.1 recipient mice were sublethal irradiated (6.0 Gy, CP-160 Faxitron) 1 day before transplantation. *lncIrf8* promoter KO and control HoxB8 MPP (single-cell clones) were expanded in vitro as described above. Cells were injected into recipient mice via the tail vein (5 million cells in 100 μl PBS per mouse). Donor cells from bone marrow and spleen were subjected to flow cytometry analysis at 7 and 14 days after cell transplantation.

### RT-qPCR

RNA was isolated by using the NucleoSpin RNA kit (Macherey-Nagel, 740955.250) according to the manufacturer’s instructions. RNA was subjected to cDNA synthesis using the High-Capacity cDNA Reverse Transcription Kit (Applied Biosystems, 4368814) with Murine RNase Inhibitor (New England Biolabs, M0314S) (Appendix 1-key resources table). RT-qPCR was performed by a StepOnePlus Real-Time PCR system (Applied Biosystems) with SYBR-green fluorescence (Applied Biosystems, 4385610). The primers for qPCR were listed in Appendix 1-key resources table. Mouse *GAPDH* was used for normalization of *lncIrf8* and *Irf8* gene expression.

### Identification of *lncIrf8*

De-novo transcript assembly of RNA-seq data was used to search for unknown transcripts with no coding potential and this identified the pDC specific lncRNA *Tcons_00190250* (referred to as *lncIrf8*) and the cDC1-specific lncRNA *Tcons_00190258*. In brief, paired end 2×100 bps reads from RNA-seq of MPP, CDP, cDC1, cDC2, and pDC were aligned to mm9 genome using Star aligner (version 2.4) (Dobin et al., 2013) and run for Cufflinks (version 2.0; Trapnell et al., 2012). Data were subjected to lenient filtering with the parameters: min isoform fraction 0.1% and pre-RNA-fraction of 0.1%, and ribosomal genes were also filtered. Next, all the predicted transcripts were merged with cuffmerge and all transcripts with no overlap with known transcripts in mouse GENCODE were selected for further analysis (Frankish et al., 2019). The coding potential and conservation of coding elements of the lncRNAs were checked with CPAT (Wang et al., 2013) and PhyloCSF (Lin et al., 2011), respectively. *lncIrf8* (*Tcons_00190250*) and *Tcons_00190258* show low scores in both analyses, which faithfully supports their role as non-coding genes and exhibit low cross-species conservation.

To further characterize the major transcripts of *lncIrf8*, 3' and 5' end Rapid Amplification of cDNA Ends (RACE) PCR was performed using template-switching RT enzyme mix (New England Biolabs, M0466) and TA cloning kit (Thermo Fisher Scientific, K202020) (Appendix 1-key resources table). The primers (listed in Appendix 1-key resources table) used for RACE PCR were synthesized by Eurofins Genomics except for 5' RACE-TSO, which was from IDT.

### 3' RACE PCR

Reverse transcript (RT) and template-switching: 4 μl (10 ng to 1 μg) total RNA (from DC differentiation day 5) were incubated at 80°C for 3 min and cooled rapidly on ice. RNA was then incubated with template-switching RT buffer, 1 mM dNTP, 5 mM DTT, 10 μM QT primer (Scotto-Lavino et al., 2006) and 1 μl RT enzyme in 10 μl at room temperature for 5 min, followed by 1 hr at 42°C, 10 min at 50°C, and 85°C for 5 min to inactive the RT enzyme mix and sample was then kept at 4°C and diluted with 20 μl MilliQ water.

First-round PCR: 1 μl of diluted sample was subjected to the first round PCR with Q0 primer and 3' RACE-GSP-*lncIrf8*-F1 (10 μM) using Q5 high fidelity DNA polymerase. Second-round PCR: the PCR products from the first round PCR (1:20 dilution) was then used as template for the second round of PCR by using Q1 primer (10 μM), 3' RACE-GSP-*lncIrf8*-F2 (10 μM). Q0 and Q1 primers and the PCR programs are described in Scotto-Lavino et al., 2006. Products from the second-round PCR were purified using the PCR clean-up kit (Macherey-Nagel*,* 740609.50).

A-tailing and TA cloning: a reaction containing 5 μl of PCR clean-up product in Taq buffer, 1 mM MgCl_2_, 0.4 mM dATP and 1 μl Taq DNA polymerase in 25 μl was prepared and incubated at 70°C for 20 min for A-tailing of PCR products. Five μl of the A-tailed products were subjected to TA cloning into pCR2.1 vector according to the manufacturer’s instruction followed by Sanger sequencing.

### 5' RACE PCR

In order to identify the TSS of *lncIrf8*, template RNA from bone marrow cell-derived pDC was used to perform 5' RACE PCR using template-switching RT enzyme mix (New England Biolabs, M0466) according to the manufacturer’s instructions. Template switching was by 5' RACE-GSP-*lncIrf8*-R2 primer (10 μM) and template switching oligo (TSO) (75 μM). Similar to the 3' RACE PCR, two rounds of PCR were used to improve PCR specificity. In brief, 5' RACE-GSP-*lncIrf8*-R2 (10 μM) and TSO-specific primer (10 μM) were used to perform the first-round PCR, 5' RACE-GSP-*lncIrf8*-R1 primer (10 μM) and the TSO-specific primer (10 μM) were used to perform the second round PCR, followed by fragments A-tailing, TA cloning, and Sanger sequencing as described above for 3' RACE PCR.

### Identification of transcription factor binding sites

Transcription factor binding sites (TFBS) in the *Irf8* +32 kb enhancer were predicted using a motif matching tool based on the MOODS (Korhonen et al., 2009) and position weight matrixes (PWMs) were obtained from the JASPAR database (Fornes et al., 2020). The bit-score cut-off thresholds were determined by applying the dynamic programming approach as described in Wilczynski et al., 2009 with an FPR of 0.0001. DC TF were considered and are depicted.

### Statistical analysis

Statistical analyses were performed using Prism (GraphPad). Unpaired t test and Multiple t-tests were used to compare data from two groups, two-way ANOVA with Tukey’s multiple comparisons test was used to analyze data from more than two groups.

## Data Availability

ATAC-seq (MPP, CDP and cDC2), Capture-C targeting *Irf8* promoter (MPP, CDP, cDC1, cDC2 and pDC), IRF8 ChIP-seq (cDC and pDC), and RNA-seq (MPP, CDP, cDC1, cDC2 and pDC) data generated in this study have been deposited in Gene Expression Omnibus and are accessible through GEO Series accession numbers GSE198651.ATAC-seq data of cDC1 and pDC (GSE118221) are published (Lin et al., 2015), and the ATAC-seq data of MPP, CDP and cDC2 of the same study are described here (GSE198651). ChIP-seq of CTCF in DC (GSE36099) (Garber et al., 2012), H3K27ac (GSE73143) (Allhoff et al., 2016), H3K4me1 and PU.1 (GSE57563) (Allhoff et al., 2014), H3K4me3 and H3K9me3 (GSE64767) (Lin et al., 2015) in MPP, CDP, cDC and pDC were re-analyzed in this study. scRNA-seq data were reanalyzed from GSE114313 (Rodrigues et al., 2018). Bulk RNA-seq data of splenic pDC, cDC1 and cDC2 were reanalyzed from GSE188992 (Pang et al., 2022).The sequence of the pDC specific lncRNA (*lncIrf8* identified by RACE PCR) and the cDC1 specific lncRNA (*Tcons_00190258* identified by RNA-seq) has been submitted to GenBank. The GenBank accession numbers for *lncIrf8* and *Tcons_00190258* are ON134061 and ON134062, respectively. The following datasets were generated: XuH LiZ KuoC GötzK LookT ToledoMA SeréK CostaIG ZenkeM 2023A lncRNA identifies Irf8 enhancer element in negative feedback control of dendritic cell differentiationNCBI Gene Expression OmnibusGSE19865110.7554/eLife.83342PMC1004254636916882 XuH LiZ KuoCC GoetzK LookT de ToldeoMAS SereK CostaIG ZenkeM 2022Mus musculus lncIRF8 lncRNA, partial sequenceNCBI NucleotideON134061 XuH LiZ KuoCC GoetzK LookT de ToldeoMAS SereK CostaIG ZenkeM 2022Mus musculus TCONS_00190258 lncRNA, partial sequenceNCBI NucleotideON134062 The following previously published datasets were used: Li et al 2019Identification of Transcription Factor Binding Sites using ATAC-seqNCBI Gene Expression OmnibusGSE11822110.1186/s13059-019-1642-2PMC639178930808370 Garber et al 2012A high throughput in vivo protein-DNA mapping approach reveals principles of dynamic gene regulation in mammals (ChIP-Seq)NCBI Gene Expression OmnibusGSE3609910.1016/j.molcel.2012.07.030PMC387310122940246 Allhoff et al 2016Differential Peak Calling of ChIP-Seq Signals with Replicates with THORNCBI Gene Expression OmnibusGSE7314310.1093/nar/gkw680PMC517534527484474 Allhoff et al 2014Detecting differential peaks in ChIP-seq signals with ODINNCBI Gene Expression OmnibusGSE5756310.1093/bioinformatics/btu72225371479 Lin et al 2015Epigenetic Program and Transcription Factor Circuitry of Dendritic Cell DevelopmentNCBI Gene Expression OmnibusGSE6476710.1093/nar/gkv1056PMC478775326476451 Rodrigues et al 2018A distinct lineage of origin reveals heterogeneity of plasmacytoid dendritic cells II (scRNAseq)NCBI Gene Expression OmnibusGSE114313 Pang et al 2022Bulk RNAseq of Mouse Splenic Dendritic Cell SubsetsNCBI Gene Expression OmnibusGSE188992

## Additional files

MDAR checklist

## Appendix 1

**Appendix 1—key resources table:**

Reagent type (species) or resource	Designation	Source or reference	Identifiers	Additional information
Genetic reagent (*M. musculus*)	C57BL/6 mice (CD45.1 and CD45.2)	Jackson Laboratory		
Genetic reagent (*M. musculus*)	Mx-Cas9-GFP mice	Medical Faculty, RWTH Aachen University Xu et al., 2022		
Cell line (*H. sapiens*)	HEK293T	ATCC	https://www.atcc.org	Lentivirus and retrovirus production
Cell line (*M. musculus*)	*lncIrf8* promoter KO HoxB8 MPP; Control HoxB8 MPP	This paper		*lncIrf8* promoter KO
Cell line (*M. musculus*)	p*lncIrf8*-pA HoxB8 MPP; pGFP-pA HoxB8 MPP	This paper		*lncIrf8* overexpression and rescue
Cell line (*M. musculus*)	*Irf8*-VP64 HoxB8 MPP; *lncIrf8*-VP64 HoxB8 MPP; Non-targeting-VP64-1 HoxB8 MPP; Non-targeting-VP64-2 HoxB8 MPP	This paper		*Irf8* and *lncIrf8* promoters activation
Cell line (*M. musculus*)	*Irf8*-KRAB HoxB8 MPP; *lncIrf8*-KRAB HoxB8 MPP; Non-targeting-KRAB-1 HoxB8 MPP; Non-targeting-KRAB-2 HoxB8 MPP	This paper		*Irf8* and *lncIrf8* promoters repression
Antibody	APC/Cyanine 7 anti-mouse/human B220 (rat monoclonal)	Biolegend	Cat# 103223; RRID: AB_313006	FACS (1:400)
Antibody	Brilliant Violet 510 anti-mouse/human CD11b (rat monoclonal)	Biolegend	Cat# 101245; RRID: AB_2561390	FACS (1:400)
Antibody	PE/Cyanine 7 anti-mouse CD11c (Armenian hamster monoclonal)	Biolegend	Cat# 117317; RRID: AB_493569	FACS (1:400)
Antibody	APC anti-mouse CD115 (rat monoclonal)	eBioscience	Cat# 17-1152-80; RRID: AB_1210789	FACS (1:400)
Antibody	PE/Cyanine 7 anti-mouse CD117 (rat monoclonal)	eBioscience	Cat# 25-1172-82; RRID: AB_469646	FACS (1:400)
Antibody	PE anti-mouse CD135 (rat monoclonal)	eBioscience	Cat# 12-1351-82; RRID: AB_465859	FACS (1:400)
Antibody	Biotin anti-mouse CD19 (rat monoclonal)	Biolegend	Cat# 115503; RRID: AB_313638	FACS (1:800)
Antibody	Biotin anti-mouse CD3e (Armenian hamster monoclonal)	eBioscience	Cat# 13-0031-82; RRID: AB_466319	FACS (1:800)
Antibody	APC/Cyanine 7 anti-mouse CD45.2 (mouse monoclonal)	Biolegend	Cat# 109823; RRID: AB_830788	FACS (1:400)
Antibody	Biotin anti-mouse F4/80 (rat monoclonal)	Biolegend	Cat# 123105; RRID: AB_893499	FACS (1:800)
Antibody	Alexa Fluor 700 anti-mouse Gr1 (rat monoclonal)	Biolegend	Cat# 108421; RRID: AB_493728	FACS (1:400)
Antibody	PerCP/Cyanine 5.5 anti-mouse Gr1 (rat monoclonal)	eBioscience	Cat# 45-5931-80; RRID: AB_906247	FACS (1:400)
Antibody	Alexa Fluor 700 anti-mouse Ly6C (rat monoclonal)	Biolegend	Cat# 128023; RRID: AB_10640119	FACS (1:400)
Antibody	Biotin anti-mouse Ly6G (rat monoclonal)	Biolegend	Cat# 127603; RRID: AB_1186105	FACS (1:800)
Antibody	Brilliant Violet 785 anti-mouse MHCII (rat monoclonal)	Biolegend	Cat# 107645; RRID: AB_2565977	FACS (1:2000)
Antibody	Biotin anti-mouse NK1.1 (mouse monoclonal)	eBioscience	Cat# 14-5941-82; RRID: AB_467736	FACS (1:800)
Antibody	Super Bright anti-mouse Siglec-H (rat monoclonal)	Invitrogen	Cat# 63-0333-82 RRID: AB_2784853	FACS (1:400)
Antibody	PE/Dazzle 594 Streptavidin	Biolegend	Cat# 405247	FACS (1:1000)
Antibody	Biotin anti-mouse Ter119 (rat monoclonal)	eBioscience	Cat# 14-5921-82; RRID: AB_467727	FACS (1:800)
Antibody	Brilliant Violet 421 anti-mouse/rat XCR1 (mouse monoclonal)	Biolegend	Cat# 148216; RRID: AB_2565230	FACS (1:400)
Antibody	7-Aminoactinomycin D (7-AAD)	Thermo Fisher Scientific	Cat# A1310	FACS (3 μl per test)
Chemical compound, drug	β-estradiol (E2)	Sigma-Aldrich	Cat# E2758	
Chemical compound, drug	β-mercaptoethanol (β-ME)	Gibco	Cat# 31350010	
Chemical compound, drug	BsmBI-v2	New England Biolabs	Cat# R0739S	
Chemical compound, drug	Chondroitin sulfate sodium salt from shark cartilage (CSS)	Sigma-Aldrich	Cat# C4384	
Chemical compound, drug	cOmplete Mini	Roche	Cat# 11836153001	
Chemical compound, drug	dATP	New England Biolabs	Cat# N0440S	
Chemical compound, drug	Dimethysulfoxide (DMSO)	Sigma-Aldrich	Cat# D8418	
Chemical compound, drug	Doxycycline hyclate	Sigma-Aldrich	Cat# D9891	
Chemical compound, drug	DpnII	A kind gift from A. Marieke Oudelaar, Max Planck Institute for Multidisciplinary Sciences, Göttingen, Germany. DpnII enzyme with a similar activity is also available from New England Biolabs.	Cat# R0543M	
Chemical compound, drug	DMEM	Gibco	Cat# 41965039	
Chemical compound, drug	DTT	Thermo Fisher Scientific	Cat# 20290	
Chemical compound, drug	EDTA	Gibco	Cat# 15575–038	
Chemical compound, drug	Fetal calf serum (FCS)	PAA	Cat# A01125-499	
Chemical compound, drug	Fetal calf serum (FCS)	Gibco	Cat# 10270106	
Chemical compound, drug	Formaldehyde (37%)	AppliChem	Cat# A0877	
Chemical compound, drug	Recombinant human Flt3-Ligand (Flt3L)	Peprotech	Cat# 300–19	
Chemical compound, drug	Recombinant murine stem cell factor (SCF)	Peprotech	Cat# 250–03	
Chemical compound, drug	Human IGF-1 long range	Sigma-Aldrich	Cat# 85,580 C	
Chemical compound, drug	Recombinant IL-6/soluble IL-6 receptor fusion protein	A kind gift from S. Rose-John, Kiel, Germany Fischer et al., 1997. R&D Systems provides a similar product with the same activity.	Cat# 9038 SR	
Chemical compound, drug	HEPES	Sigma-Aldrich	Cat# H4034	
Chemical compound, drug	L-glutamine	Gibco	Cat# 25030081	
Chemical compound, drug	M-270 Streptavidin Dynabeads	Invitrogen	Cat# 65305	
Chemical compound, drug	Mouse interferon α (mIFNα)	Miltenyi Biotec	Cat# 130-093-131	
Chemical compound, drug	Murine RNase Inhibitor	New England Biolabs	Cat# M0314S	
Chemical compound, drug	Pancoll human, density 1.077 g/ml (Ficoll)	PAN-Biotech	Cat# P04-601000	
Chemical compound, drug	Penicillin/streptomycin	Gibco	Cat# 15140122	
Chemical compound, drug	Phenol-Chloroform-Isoamyl alcohol (PCI)	Sigma-Aldrich	Cat# 77617	
Chemical compound, drug	Phosphate buffered saline (PBS)	Gibco	Cat# 10010023	
Chemical compound, drug	Polybrene (PB, Hexadimethrine bromide)	Sigma-Aldrich	Cat# H9268	
Chemical compound, drug	Q5 high fidelity DNA polymerase	New England Biolabs	Cat# M0491L	
Chemical compound, drug	RPMI 1640	Gibco	Cat# 31870025	
Chemical compound, drug	SalI	New England Biolabs	Cat# R0138S	
Chemical compound, drug	Supernatant from Flt3L-producing B16F1 cells (1%)	Homemade. Flt3L from Peprotech has the same activity (1:1000)	Cat# 300–19	
Chemical compound, drug	Supernatant from CHO KLS C6 cells expressing soluble murine SCF (1%)	Homemade. Peprotech provides a similar product with the same activity (1:1000).	Cat# 250–03	
Chemical compound, drug	SYBR-green fluorescence	Applied Biosystems	Cat# 4385610	
Chemical compound, drug	T4 DNA HC ligase	Life Tech	Cat# EL0013	
Chemical compound, drug	Taq DNA polymerase	Homemade		
Chemical compound, drug	Taq buffer (10 x)	Thermo Fisher Scientific	Cat# B33	
Chemical compound, drug	Template-switching RT enzyme mix	New England Biolabs	Cat# M0466	
Chemical compound, drug	XhoI	New England Biolabs	Cat# R0146S	
Commercial assay or kit	Gibson Assembly kit	New England Biolabs	Cat# E5510S	
Commercial assay or kit	High-Capacity cDNA Reverse Transcription Kit	Applied Biosystems	Cat# 4368814	
Commercial assay or kit	KAPA Hyper Capture Reagent Kit	Roche	Cat# 9075828001	
Commercial assay or kit	NEBNext Ultra II DNA Library Prep Kit for Illumina	New England Biolabs	Cat# E7645S	
Commercial assay or kit	NEBNext Multiplex Oligos for Illumina (Index Primers Set 1)	New England Biolabs	Cat# E7335S	
Commercial assay or kit	NEBNext Multiplex Oligos for Illumina (Index Primers Set 2)	New England Biolabs	Cat# E7500S	
Commercial assay or kit	NucleoSpin RNA kit	Macherey-Nagel	Cat# 740955.250	
Commercial assay or kit	PCR clean-up kit	Macherey-Nagel	Cat# 740609.50	
Commercial assay or kit	TA cloning kit	Thermo Fisher Scientific	Cat# K202020	
Sequence-based reagent	5'RACE-TSO	New England Biolabs	5’ RACE PCR primers	GCTAATCATTGCAAGCAGTGGTATC AACGCAGAGTACATrGrGrG
Sequence-based reagent	5'RACE-TSO-Specific	New England Biolabs	5’ RACE PCR primers	CATTGCAAGCAGTGGTATCAAC
Sequence-based reagent	5'RACE-GSP-*lncIrf8*-R1	New England Biolabs	5’ RACE PCR primers	TGTCAGTGATGGGGGCTGGAGAAAT
Sequence-based reagent	5'RACE-GSP-*lncIrf8*-R2	New England Biolabs	5’ RACE PCR primers	GCTCAGGATGCCAGGTCCCTTCTT
Sequence-based reagent	3'RACE-QT	Scotto-Lavino et al., 2006	3’ RACE PCR primers	CCAGTGAGCAGAGTGACGAGGACTC GAGCTCAAGCTTTTTTTTTTTTTTTTT
Sequence-based reagent	3'RACE-Q0	Scotto-Lavino et al., 2006	3’ RACE PCR primers	CCAGTGAGCAGAGTGACG
Sequence-based reagent	3'RACE-QI	Scotto-Lavino et al., 2006	3’ RACE PCR primers	GAGGACTCGAGCTCAAGC
Sequence-based reagent	3'RACE-GSP-*lncIrf8*-F1	This paper	3’ RACE PCR primers	ATTTCTCCAGCCCCCATCACTGACA
Sequence-based reagent	3'RACE-GSP-*lncIrf8*-F2	This paper	3’ RACE PCR primers	AAGAAGGGACCTGGCATCCTGAGC
Sequence-based reagent	*lncIrf8*-F	This paper	Genotyping primers	TCCTGAAGGGACAGGCAAG
Sequence-based reagent	*lncIrf8*-R	This paper	Genotyping primers	CTTGGACATTGAGGACGCC
Sequence-based reagent	cDC1 +32 kb-F1	This paper	Genotyping primers	GTGACTGCAAGTAAGTTCTTCGG
Sequence-based reagent	cDC1 +32 kb-F2	This paper	Genotyping primers	AAGTAGAGATTCCCTTTCTAAGCC
Sequence-based reagent	cDC1 +32 kb-R	This paper	Genotyping primers	ATCAGGCTGGGTGGTGGTT
Sequence-based reagent	Sc-*lncIrf8*-F	This paper	Cloning primers	ACACTCGAGACTGTCAGATGCAGGGG; the underline sequences represent cloning sites
Sequence-based reagent	Sc-*lncIrf8*-R	This paper	Cloning primers	AAAAAAGTCGACGCATCAGATTTAATATA GAACTAGGACA; the underline sequences represent cloning sites
Sequence-based reagent	CMV-*lncIrf8*-F	This paper	Genotyping primers	TGGGCGTGGATAGCGGTTT
Sequence-based reagent	CMV-*lncIrf8*-R	This paper	Genotyping primers	CACTGAGACTTAGCAAGGGGGA
Sequence-based reagent	CMV-GFP-F	This paper	Genotyping primers	TGGGCGTGGATAGCGGTTT
Sequence-based reagent	CMV-GFP-R	This paper	Genotyping primers	TGGGGGTGTTCTGCTGGTAG
Sequence-based reagent	m*lncIrf8*-tQ-F	This paper	RT-qPCR primers	ACTGTCAGATGCAGGGG
Sequence-based reagent	m*lncIrf8*-tQ-R	This paper	RT-qPCR primers	TCACAATCGTCTGTAACTCCG
Sequence-based reagent	m*Irf8*-tQ-F	This paper	RT-qPCR primers	GAGCGAAGTTCCTGAGATGG
Sequence-based reagent	m*Irf8*-tQ-R	This paper	RT-qPCR primers	TGGGCTCCTCTTGGTCATAC
Sequence-based reagent	m*GAPDH*-tQ-F	This paper	RT-qPCR primers	ACCTGCCAAGTATGATGACATCA
Sequence-based reagent	m*GAPDH*-tQ-R	This paper	RT-qPCR primers	GGTCCTCAGTGTAGCCCAAGAT
Sequence-based reagent	m3C-F	Downes et al., 2021; Downes et al., 2022	Capture-C qPCR primers	GGAGAAAGAAGGCTGGTGTTAT
Sequence-based reagent	m3C-R	Downes et al., 2021; Downes et al., 2022	Capture-C qPCR primers	TATCTGAGTTGGACAGCATTGG
Sequence-based reagent	m3C-control-F	Downes et al., 2021; Downes et al., 2022	Capture-C qPCR primers	TTATCTTGCATTTGCCAACTCG
Sequence-based reagent	m3C-control-R	Downes et al., 2021; Downes et al., 2022	Capture-C qPCR primers	TGGGTTTCCCTGATTCTGAAA
Sequence-based reagent	*Irf8*_P_L	This paper	Capture probes	GATCCGTGCATCACCAGCCTCC TTGACCTTAGGCAGACGCCCCA GCCCCCCGGCCATTTTTGGGGCAGCC
Sequence-based reagent	*Irf8*_P_R	This paper	Capture probes	CCAAATGAACAAACACCTCTCCC TTTAAAATCTGCCTGATGGCCAA CTTCATAATGAAGAGAAATAGATC
Sequence-based reagent	gRNA-1-F	This paper	gRNAs	CACCGTCCATTATACTAAGATACCC; the underline sequences represent cloning sites
Sequence-based reagent	gRNA-1-R	This paper	gRNAs	AAACGGGTATCTTAGTATAATGGAC; the underline sequences represent cloning sites
Sequence-based reagent	gRNA-2-F	This paper	gRNAs	CACCGGTGCCGAGAAAGGACACGT; the underline sequences represent cloning sites
Sequence-based reagent	gRNA-2-R	This paper	gRNAs	AAACACGTGTCCTTTCTCGGCACC; the underline sequences represent cloning sites
Sequence-based reagent	gRNA-KO1-5‘F	Durai et al., 2019	gRNAs	CACCGTTGTGATCTTTGAGGTAGA; the underline sequences represent cloning sites
Sequence-based reagent	gRNA-KO1-5’R	Durai et al., 2019	gRNAs	AAACTCTACCTCAAAGATCACAAC; the underline sequences represent cloning sites
Sequence-based reagent	gRNA-KO1-3‘F	Durai et al., 2019	gRNAs	CACCGAACTGGCCTGGGGCAGGTC; the underline sequences represent cloning sites
Sequence-based reagent	gRNA-KO1-3’R	Durai et al., 2019	gRNAs	AAACGACCTGCCCCAGGCCAGTTC; the underline sequences represent cloning sites
Sequence-based reagent	gRNA-KO2-5’F	This paper	gRNAs	CACCGACATTCTGCACCCCAGTCA; the underline sequences represent cloning sites
Sequence-based reagent	gRNA-KO2-5’R	This paper	gRNAs	AAACTGACTGGGGTGCAGAATGTC; the underline sequences represent cloning sites
Sequence-based reagent	gRNA-KO2-3’F	This paper	gRNAs	CACCGAGGATCGCACCTCACCTACT; the underline sequences represent cloning sites
Sequence-based reagent	gRNA-KO2-3’R	This paper	gRNAs	AAACAGTAGGTGAGGTGCGATCCTC; the underline sequences represent cloning sites
Sequence-based reagent	gRNA-*Irf8*-VP64-F	This paper	gRNAs	CACCGACGGTCGCGCGAGCTAATTG; the underline sequences represent cloning sites
Sequence-based reagent	gRNA-*Irf8*-VP64-R	This paper	gRNAs	AAACCAATTAGCTCGCGCGACCGTC; the underline sequences represent cloning sites
Sequence-based reagent	gRNA-*Irf8*-KRAB-F	This paper	gRNAs	CACCGCGGCAGGTAGGACGCGATG; the underline sequences represent cloning sites
Sequence-based reagent	gRNA-*Irf8*-KRAB-R	This paper	gRNAs	AAACCATCGCGTCCTACCTGCCGC; the underline sequences represent cloning sites
Sequence-based reagent	gRNA-*lncIrf8*-VP64-F	This paper	gRNAs	CACCGGTGCCGAGAAAGGACACGT; the underline sequences represent cloning sites
Sequence-based reagent	gRNA-*lncIrf8*-VP64-R	This paper	gRNAs	AAACACGTGTCCTTTCTCGGCACC; the underline sequences represent cloning sites
Sequence-based reagent	gRNA-*lncIrf8*-KRAB-F	This paper	gRNAs	CACCGAGTCACTCGTCCTTTGGGG; the underline sequences represent cloning sites
Sequence-based reagent	gRNA-*lncIrf8*-KRAB-R	This paper	gRNAs	AAACCCCCAAAGGACGAGTGACTC; the underline sequences represent cloning sites
Sequence-based reagent	gRNA-non-targeting-1-F	Manguso et al., 2017	gRNAs	CACCGCGAGGTATTCGGCTCCGCG; the underline sequences represent cloning sites
Sequence-based reagent	gRNA-non-targeting-1-R	Manguso et al., 2017	gRNAs	AAACCGCGGAGCCGAATACCTCGC; the underline sequences represent cloning sites
Sequence-based reagent	gRNA-non-targeting-2-F	Manguso et al., 2017	gRNAs	CACCGATGTTGCAGTTCGGCTCGAT; the underline sequences represent cloning sites
Sequence-based reagent	gRNA-non-targeting-2-R	Manguso et al., 2017	gRNAs	AAACATCGAGCCGAACTGCAACATC; the underline sequences represent cloning sites
Software, algorithm	Bamtofastq	10 x Genomics	https://support.10xgenomics.com/docs/bamtofastq	
Software, algorithm	Bowtie2	Langmead and Salzberg, 2012	http://bowtie-bio.sourceforge.net	
Software, algorithm	CapCruncher (v0.1.1a1)	Downes et al., 2022	https://github.com/sims-lab/CapCruncher	
Software, algorithm	CPAT	Wang et al., 2013	http://code.google.com/p/cpat/	
Software, algorithm	Cufflinks (version 2.0)	Trapnell et al., 2012	http://cufflinks.cbcb.umd.edu/	
Software, algorithm	FlowJo V10	BD Biosciences		
Software, algorithm	IGV browser	Broad Institute		
Software, algorithm	MOODS	Korhonen et al., 2009	https://www.cs.helsinki.fi/group/pssmfind/	
Software, algorithm	Oligo	Oudelaar et al., 2020	https://oligo.readthedocs.io/en/latest/index.html	
Software, algorithm	PhyloCSF	Lin et al., 2011	http://compbio.mit.edu/PhyloCSF	
Software, algorithm	Prism	GraphPad		
Software, algorithm	Scanpy	Wolf et al., 2018		
Software, algorithm	Star aligner (version 2.4)	Dobin et al., 2013	http://code.google.com/p/rna-star/	
Software, algorithm	Snapgene	GSL Biotech		
Software, algorithm	UMAP	Becht et al., 2019		
