# Supplementary figures and images for: A lncRNA identifies Irf8 enhancer element in negative feedback control of dendritic cell differentiation
Source: eLife. 2023 Mar 14;12:e83342. doi: 10.7554/eLife.83342 (PMC10042546; doi:10.7554/eLife.83342)

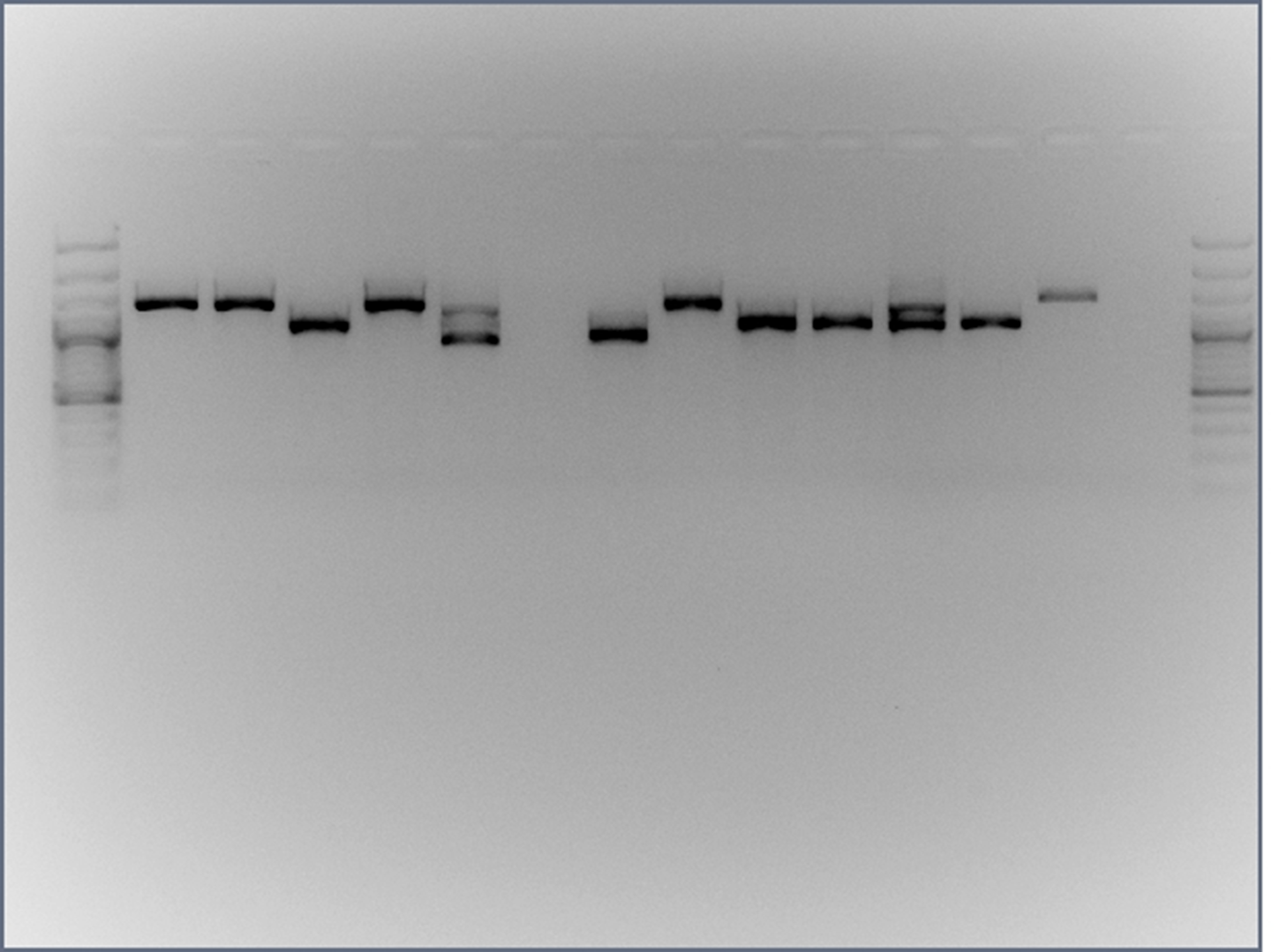

Supplement: Figure 2—figure supplement 1—source data 1. [file elife-83342-fig2-figsupp1-data1.tif]

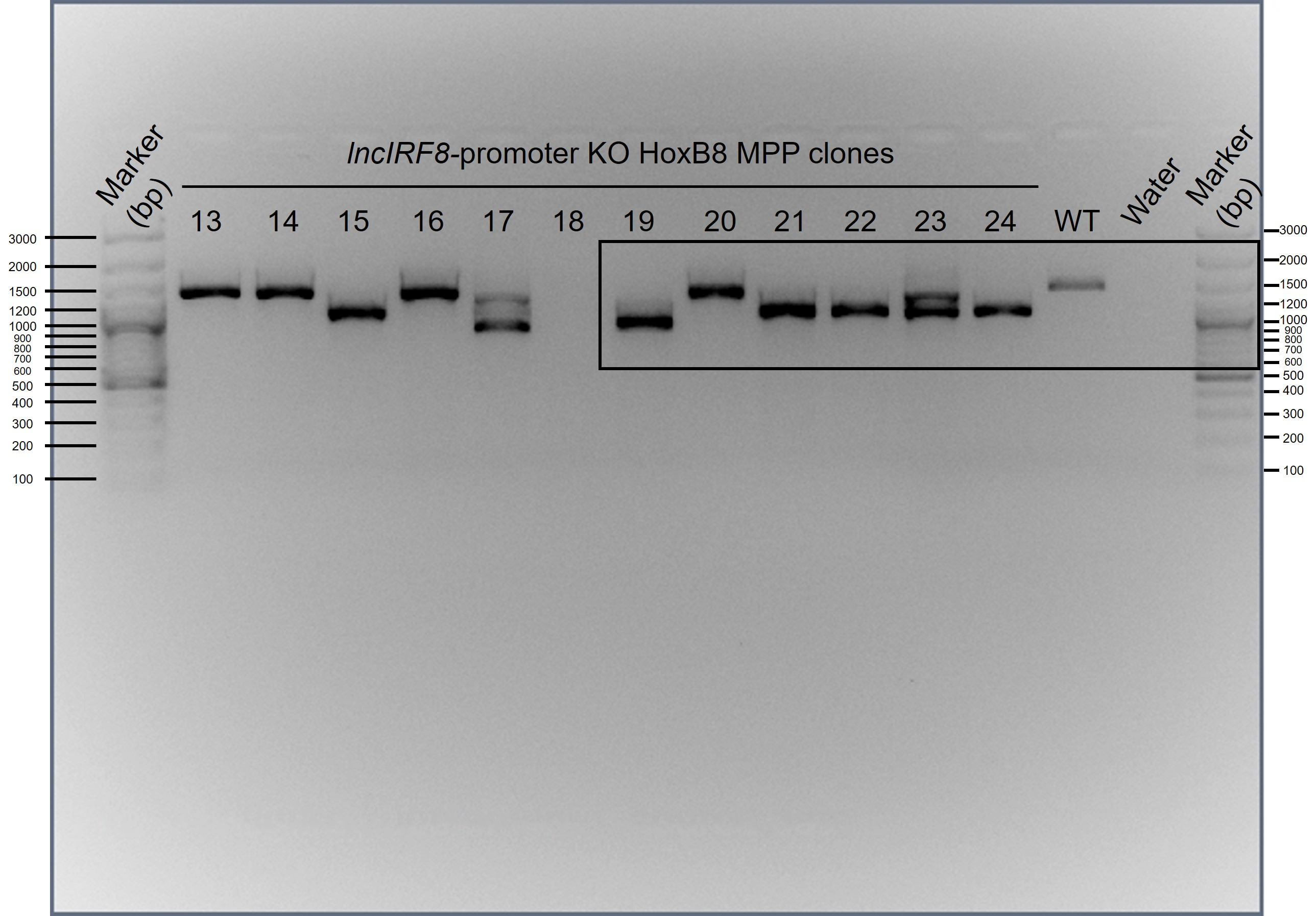

Supplement: Figure 2—figure supplement 1—source data 2. [file elife-83342-fig2-figsupp1-data2.tif]
